# Bacterial lectin BambL acts as a B cell superantigen

**DOI:** 10.1007/s00018-021-04009-z

**Published:** 2021-11-03

**Authors:** Marco Frensch, Christina Jäger, Peter F. Müller, Annamaria Tadić, Isabel Wilhelm, Sarah Wehrum, Britta Diedrich, Beate Fischer, Ana Valeria Meléndez, Joern Dengjel, Hermann Eibel, Winfried Römer

**Affiliations:** 1 Faculty of Biology, University of Freiburg, Freiburg, Germany; 2 Signaling Research Centers BIOSS and CIBSS, University of Freiburg, Freiburg, Germany; 3 International Max Planck Research School for Molecular and Cellular Biology (IMPRS-MCB), Max Planck Institute of Immunobiology and Epigenetics, Freiburg, Germany; 4 Spemann Graduate School of Biology and Medicine (SGBM), University of Freiburg, Freiburg, Germany; 5 Department of Biology, University of Fribourg, Fribourg, Switzerland; 6 Department of Dermatology, University Medical Center and University of Freiburg, Freiburg, Germany; 7 Center for Chronic Immunodeficiency, CCI and University Medical Center Freiburg, Freiburg, Germany; 8 Freiburg Institute for Advanced Studies (FRIAS), University of Freiburg, Freiburg, Germany

**Keywords:** Adaptive immunity, Bacterial pathogens, Multivalent lectins, Immunoglobulin glycosylation, β-Propeller lectins, Apoptosis

## Abstract

B cell superantigens crosslink conserved domains of B cell receptors (BCRs) and cause dysregulated, polyclonal B cell activation irrespective of normal BCR-antigen complementarity. The cells typically succumb to activation-induced cell death, which can impede the adaptive immune response and favor infection. In the present study, we demonstrate that the fucose-binding lectin of *Burkholderia ambifaria*, BambL, bears functional resemblance to B cell superantigens. By engaging surface glycans, the bacterial lectin activated human peripheral blood B cells, which manifested in the surface expression of CD69, CD54 and CD86 but became increasingly cytotoxic at higher concentrations. The effects were sensitive to BCR pathway inhibitors and excess fucose, which corroborates a glycan-driven mode of action. Interactome analyses in a model cell line suggest BambL binds directly to glycans of the BCR and regulatory coreceptors. In vitro, BambL triggered BCR signaling and induced CD19 internalization and degradation. Owing to the lectin’s six binding sites, we propose a BCR activation model in which BambL functions as a clustering hub for receptor glycans, modulates normal BCR regulation, and induces cell death through exhaustive activation.

**Supplementary Information:**

The online version contains supplementary material available at 10.1007/s00018-021-04009-z.

## Introduction

Classically, naive B cells are activated by matching antigens that crosslink the unique complementarity-determining regions (CDRs) of B cell receptors (BCRs). This specific interaction of CDR and cognate antigen is at the heart of adaptive immunity, as it sets a carefully regulated cellular differentiation program in motion and generates antibodies tailored specifically to the previously recognized antigen. B cell superantigens, in contrast, target and crosslink conserved immunoglobulin (Ig) sites outside the CDRs, circumventing BCR specificity and causing a malignant polyclonal activation of B cells (reviewed in [1]). Staphylococcal protein A (SpA) is the best-studied example [2, 3], but several more bacterial [4–10], viral [11–13] and eukaryotic [14] B cell superantigens have been described. Typically, these agents induce BCR capping and expression of classical surface activation markers (including CD69, CD86 and CD54), followed by activation-induced cell death (AICD) through caspase-dependent apoptosis [1, 3, 15, 16]. Some studies also reported a mitogenic effect of B cell superantigens [17–21], but this aspect has been controversially debated [1]. However, there is consensus in the literature that superantigens can severely confound humoral immunity and dampen the clearance of infections [1, 3, 22].

**Fig. 1:**
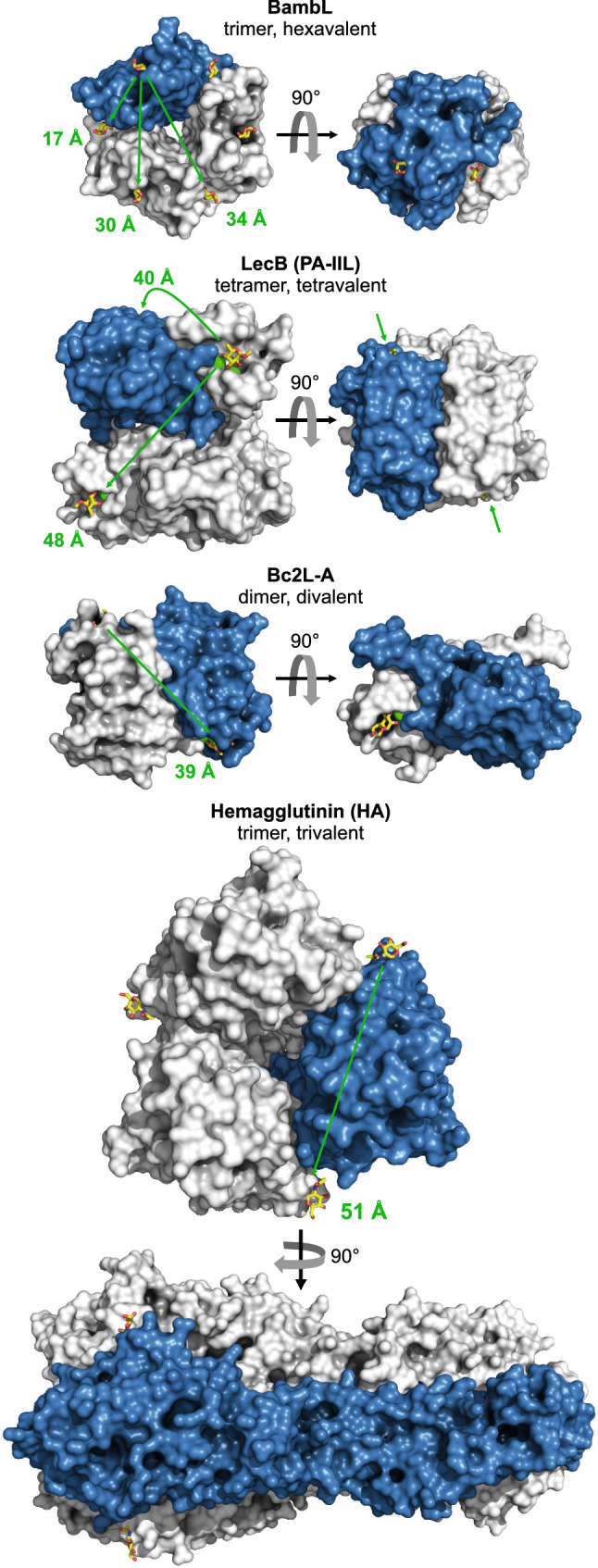
Structure comparison of B cell-activating and BCR-binding lectins. BambL (from *Burkholderia ambifaria*, PDB entry 3ZWE), LecB (*Pseudomonas aeruginosa*, 5A6X), Bc2L-A (*B. cenocepacia*, 4AOC), and hemagglutinin (H5N1 influenza virus, 2FK0). Proteins are rendered with PyMOL as surface presentations, sugars as sticks, and calcium ions in the binding pockets of LecB and Bc2L-A as green spheres. One monomer per lectin is colored blue. Arrows and numbers mark the approximate distances between individual sugar binding sites. BambL, although the smallest in size, has by far the highest valency

B cell superantigens rely on conserved Ig protein domains, but four structurally distinct, multivalent lectins (Fig. 1) have been found to exert similar effects by engaging the glycans conjugated to BCR-Igs: LecB from *Pseudomonas aeruginosa* and Bc2L-A from *Burkholderia cenocepacia* [23], hemagglutinin from influenza virus [24], and the *Burkholderia ambifaria* lectin BambL, which we have recently demonstrated to possess potent superantigenicity toward murine B cells [25]. The trimeric, hexavalent, fucose-binding lectin elicited polyclonal activation and deletion of B cells in vivo, which was accompanied by splenomegaly. Knockout of the IgD-type BCR, the coreceptor CD19 or the kinase SYK abolished the reaction in isolated B cells ex vivo, which suggests BCR signaling to constitute the major pathway through which BambL activates these cells. Herein, the lectin’s high avidity and potential to crosslink receptors may play an important mechanistic part, but predicting molecular interactions is difficult based on currently available data for receptor glycans. Understanding the mechanistic role of the BCR as a gateway to glycan-mediated B cell activation is still at an early stage, even though lymphocyte stimulation by lectins has long been known [26].

The extent to which lectins contribute to bacterial pathogenicity is still an expanding area of research. While lectins are only one of many virulence factors, these proteins can induce a multitude of effects despite being devoid of catalytic activity. By binding to glycoproteins and -lipids, lectins act not only as adhesins but also induce receptor clustering and internalization, deform membranes, create pores, and overall, disturb normal membrane organization and cell signaling [27–31]. Fucose-binding, tetravalent LecB is known to convey bacterial cell–cell and cell–matrix adhesion, but recent studies in our group have demonstrated that the isolated lectin can also trigger cellular degradation of integrin and growth-factor receptors, impair wound healing and may thereby foster chronic infections [32–34]. In another study, LecB was implicated in contributing to alveolar capillary tissue damage and bacterial dissemination [35]. It appears feasible that hexavalent BambL can exert similar effects. Incidentally, humans suffering from cystic fibrosis exhibit increased surface fucosylation of lung tissue [36, 37]—and are especially susceptible to *Pseudomonas* and *Burkholderia* infections [38]. Notably, although *B. ambifaria* is one of the less frequent species prevailing in clinical isolates [39], BambL’s β-propeller structure is a common lectin fold recurring across all domains of life [40]. Computational analyses predicted similar six-bladed lectins in the genomes of around 200 bacterial species, and seven-bladed lectins in about 2500 [41].

In the present study, we investigated the cellular responses of human B cells to BambL exposure. BambL achieved polyclonal activation of peripheral blood B cells at low nanomolar concentrations, which manifested in the expression of activation markers reminiscent of a classical, cognate BCR stimulation. Naive B cells responded more strongly than other B cell subsets, and higher concentrations of lectin proved cytotoxic. Our interactome studies with the Ramos B cell line suggest BambL binds directly to the BCR and regulatory coreceptors. In vitro exposure to BambL stimulated members of the intracellular BCR signaling pathway, and provoked internalization and degradation of CD19, but not of the IgM-type BCR. In conclusion, we propose BambL functions as a multivalent clustering hub on B cell membranes and modulates naive receptor organization, triggers intracellular signaling, and pan-activates B cells irrespective of antigen complementarity similar to classical B cell superantigens.

## Materials and methods

### BambL production and labeling

Transformed *E. coli* BL21 (DE3) harboring a pET25-BambL plasmid with a codon-optimized nucleotide sequence of BambL from *B. ambifaria* AMMD (UniProt ID Q0B4G1, 9.38 kDa per monomer) were kindly provided by Dr. Anne Imberty (CNRS, Univ. of Grenoble, France). BambL was recombinantly produced and purified as described before [42]. Briefly, bacteria in logarithmic growth phase were supplemented with 0.5 mM IPTG and cultivated for 16 h at 18 °C. Cells were collected by centrifugation, resuspended in column equilibration buffer (20 mM Tris pH 7.5, 1 M NaCl) and lysed with a French press cell disruptor. The lysate was cleared by centrifugation and subjected to affinity column purification using an Äkta FPLC system (GE Healthcare), equipped with a 5-mL column of D-mannosylated agarose (Merck). BambL was eluted in 20 mM Tris, pH 8.8 and 50 mM D-mannose, and dialyzed in SnakeSkin Tubing of 10k MW cutoff (Thermo Fisher) against water for 7 days at 4 °C to remove the sugar. The lectin solution was concentrated in centrifugation filters and sterile-filtered. Purity was confirmed by SDS-PAGE using a Coomassie blue staining, as well as by western blot using an antibody raised against the lectin’s C terminus (Eurogentec). Size-exclusion chromatography, using a HiLoad 26/600 Superdex 200 pg column (GE Healthcare), yielded a single peak at the expected retention time and indicated BambL to have assumed its proper trimeric fold.

Biotinylated BambL (BambL-biotin) was generated with the EZ-Link Sulfo-NHS-SS-biotinylation kit (Thermo Fisher), using a 20 × molar excess of the biotin ester and 2 h reaction time at 4 °C. Labeling efficiency was evaluated with the HABA assay, yielding 3 biotin moieties per functional BambL trimer. Fluorescent BambL for the 700-nm channel of lectin blots (BambL-700) was generated with the IRDye 680RD protein labeling kit for low molecular weights (Li-Cor), using a 10 × molar excess of the dye ester and 3 h reaction time at RT. Fluorescent BambL for microscopy (BambL-488) was generated with Alexa Fluor 488 NHS-Ester (Thermo Fisher), using a 5 × molar excess of the dye ester and 2 h reaction time at RT. For both fluorescent lectins, the labeling efficiency was evaluated photometrically, yielding 2–3 dye moieties per BambL trimer.

### Cell isolation and cultivation

Human peripheral blood mononuclear cells (PBMCs) were enriched from blood of healthy donors by Ficoll density gradient centrifugation. CD19^+^ cells (B cells) were freshly isolated from PBMCs using either the MojoSort (Biolegend) or EasySep (Stem Cell Tech) B cell isolation kit by means of negative selection with magnetic cell sorting (MACS), plated at 5 × 10^4^–10^6^ cells/well and allowed to rest for 1 h prior to stimulation experiments. Primary cells were cultivated in IMDM supplemented with 10% FCS (Thermo Fisher). Ramos and BJAB B cells were cultivated in RPMI 1640 supplemented with 10% FCS, 1% L-glutamine, 100 U/mL penicillin and 100 µg/mL streptomycin (Thermo Fisher).

### Flow-cytometric analyses

After stimulation, live cells were transferred into cold FACS buffer (PBS, 5% FCS, 0.5 mM EDTA). Antibody staining was carried out according to the manufacturers’ recommendations, followed by a brief staining with DAPI to mark non-viable cells. Antibodies for flow cytometry were purchased from Biolegend (CD19: clone HIB19 catalogue #302218 and #302233; CD22: clone HIB22 #302517; CD27: clone O232 #302820; CD45: clone HI30 #304020; CD54: clone HCD54 #322718; CD69: clone FN50 #310906; CD79b: clone CB3-1 #341404; CD80: clone 2D10 #305216; IgM: clone MHM-88 #314506), BD Biosciences (CD86: clone 2331/FUN-1, #555658) or Southern Biotech (IgD: polyclonal #2030-02; IgM: polyclonal #2022-01). Samples were analyzed with a Canto II (Becton Dickinson) or Attune NxT (Thermo Fisher) flow cytometer.

### Primary B cell activation and survival (Fig.2)

**Fig. 2:**
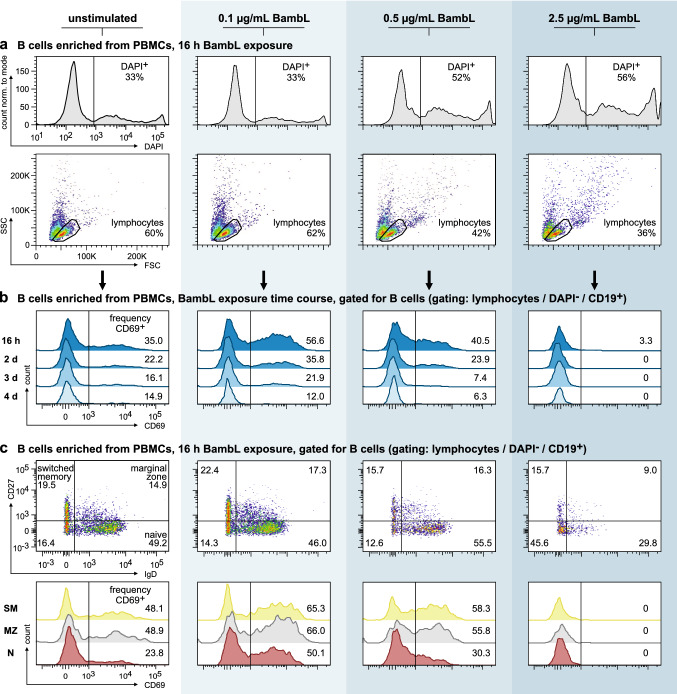
BambL activates peripheral human B cells but becomes cytotoxic at increasing concentrations. B cells were enriched from peripheral blood mononuclear cells (PBMCs) and cultivated in the presence of three different BambL concentrations or left unstimulated for 4 days (*n* = 1 for each data point). Samples were analyzed by flow cytometry after each day. **a** BambL is cytotoxic to B cells. Histograms of DAPI fluorescence (first row) and light scattering plots (second row) of ungated cells after 16 h of lectin exposure demonstrate that 0.1 µg/mL BambL is tolerated well, but higher concentrations become increasingly cytotoxic. **b** BambL stimulates surface CD69 expression. Viable B cells (DAPI^−^ CD19^+^) were identified in the lymphocytes gate in a and analyzed for surface CD69 fluorescence. Histograms depict the time course over 4 days. **c** All B cell subsets are affected by BambL. Viable naive (‘N’, IgD^+^ CD27^−^), marginal‑zone (‘MZ’, IgD^+^ CD27^+^) and class-switched memory (‘SM’, IgD^−^ CD27^+^) B cells were identified in samples after 16 h lectin exposure (scatter plots in first row, numbers in quadrants represent subset proportions). Surface CD69 fluorescence intensities are presented as histograms in the second row. While all subsets experienced an increase in surface CD69, the naive cells displayed the strongest response

Purified B cells from a single donor were collected by centrifugation, resuspended in stimulation media with 0, 0.1, 0.5 or 2.5 µg/mL recombinant BambL (corresponding to 3.6, 17.8 or 90 nM functional lectin trimers) and monitored for their activation for 4 days (first time point after 16 h). Individual wells were prepared for each time point and stimulation condition (*n* = 1 for each data point). For analysis, cells were collected by centrifugation and stained with antibodies against CD19, IgD, CD27 and CD69. The frequencies of DAPI-positive cells were calculated from the whole, ungated samples. However, only the viable B cells therein were considered in the analysis of CD69 expression. Subsets were defined as IgD^+^ CD27^−^ (naive), IgD^+^ CD27^+^ (marginal zone) and IgD^−^ CD27^+^ (class-switched memory B cells).

### Analysis of surface activation markers (Fig. 3a)

**Fig. 3:**
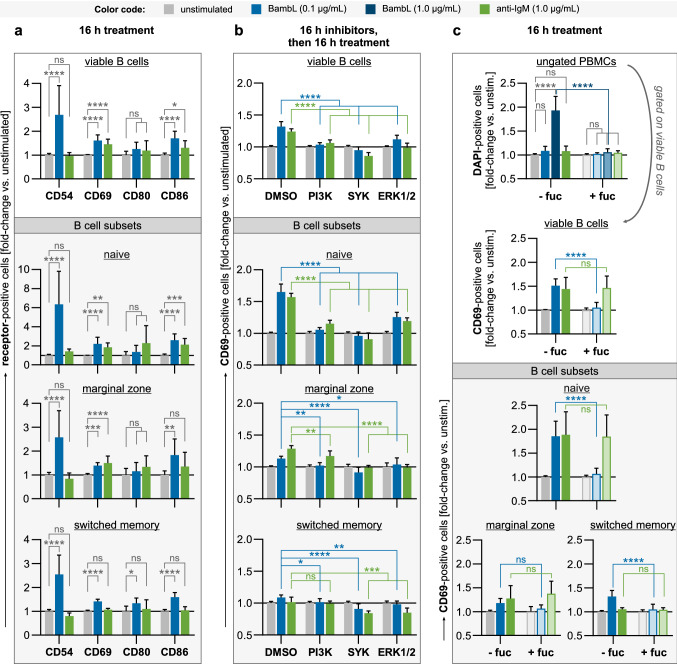
BambL stimulates the expression of classical activation markers, which is sensitive to inhibition of SYK, PI3K and ERK1/2. PBMCs were treated overnight and analyzed by flow cytometry. Viable B cells (DAPI^−^ CD19^+^) and B cell subsets were identified within the samples after analysis as before. Bars are mean fold changes of signal-positive cell frequencies relative to unstimulated controls (error bars are SD). Statistical significance (ANOVA with Tukey’s correction): ns (*p* > 0.05), * (*p* ≤ 0.05), ** (*p* ≤ 0.01), *** (*p* ≤ 0.001), **** (*p* ≤ 0.0001). **a** BambL stimulates the expression of B cell activation markers. PBMCs (*n* = 9–10) were cultivated for 16 h in the presence of BambL, anti-IgM or left unstimulated. BambL elicited an upregulation of CD54, CD69 and CD86, which was most prominent in the naive B cell subset. **b** Inhibition of intracellular pathways diminishes B cell activation by BambL. B cells (*n* = 6–9) were first enriched from PBMCs and pre-treated with inhibitors against PI3K, SYK or ERK1/2 for 16 h (‘DMSO’: solvent controls), then stimulated and analyzed for surface expression of CD69 as before. **c** Excess fucose prevents cell death and activation. PBMCs (*n* = 9) were stimulated as in a, but 1 µg/mL BambL was included as an additional, cytotoxic stimulus in this experiment. A second set of treatment solutions was supplemented with L-fucose prior to the experiment (‘ + fuc’). The first panel shows the relative frequencies of all DAPI-positive cells (ungated PBMCs). Herein, viable B cells were identified and analyzed for their surface CD69 expression as before (second panel and subset panels). Samples exposed to 1 µg/mL lectin were excluded from the CD69 analyses because of their magnitude of cell death

Total PBMCs were stimulated with 0.1 µg/mL (3.6 nM) BambL or 1 µg/mL (9 nM) anti-IgM for 16 h. Control samples were left unstimulated for 16 h. Cells were stained with antibodies against activation markers CD69, CD86, CD80 and CD54, in addition to CD19, IgD and CD27. Frequencies of viable and receptor-positive B cells were calculated relative to unstimulated controls before pooling the data from individual experiments. Bars represent mean fold changes ± SD (*n* = 9–10). Statistical significance was evaluated by ANOVA with Tukey’s correction.

### Inhibition of B cell activation pathways (Fig. 3b)

Purified B cells were pre-treated with inhibitors against SYK (R406, 2.5 µM), PI3K p110γ/δ (Nemiralisib, 10 µM) or ERK1/2 (U0126, 10 µM) for 16 h. All inhibitors were purchased from Selleckchem and dissolved in DMSO. DMSO-treated cells served as controls. The cells were then collected by centrifugation, washed and resuspended in stimulation media containing either 0.1 µg/mL BambL, 1 µg/mL anti-IgM or no stimulant. After 16 h, cells were stained with antibodies against CD19, IgD, CD27 and CD69. Frequencies of viable and CD69-positive B cells were calculated relative to unstimulated controls before pooling the data from individual experiments. Bars represent mean fold changes ± SD (n = 6–9). Statistical significance was evaluated by ANOVA with Tukey’s correction.

### Fucose blocks cell death and activation (Fig. 3c)

Total PBMCs were stimulated with either BambL (0.1 or 1 µg/mL) or anti-IgM (1 µg/mL). Unstimulated cells served as negative controls. For the fucose inhibition test, a second set of treatment media was supplemented with 1 µg/mL (6 µM) L-fucose and allowed to equilibrate for 20 min prior to cell stimulation. After 16 h of stimulation, cells were stained with antibodies against CD19, IgD, CD27 and CD69 as before. The frequencies of DAPI-positive cells were calculated from the whole, ungated samples. For further analysis (CD69 expression and subsets gating), only viable B cells were considered. Because of the magnitude of cell death after stimulation with 1 µg/mL BambL, this condition was excluded from CD69 analyses. Frequencies of CD69-positive cells were calculated relative to unstimulated controls before pooling the data from individual experiments. Bars represent mean fold changes ± SD (*n* = 9). Statistical significance was evaluated by ANOVA with Tukey’s correction.

### Mass-spectrometric identification of BambL interaction partners on Ramos cells (Fig. 4a)

**Fig. 4:**
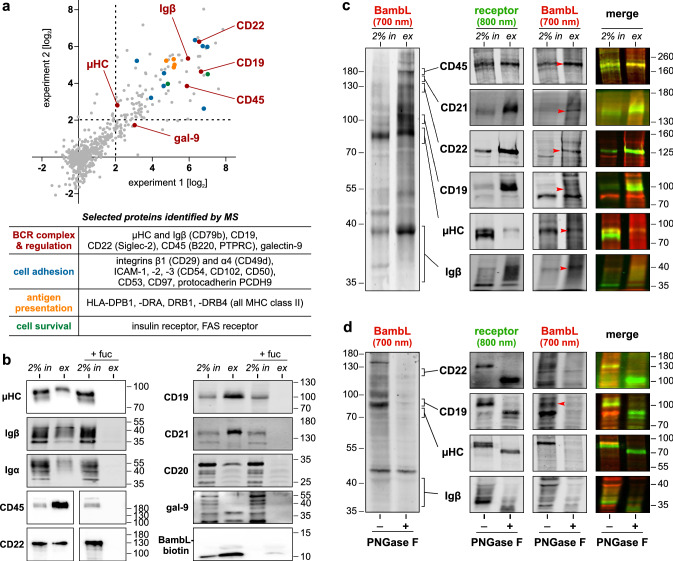
BambL extracts the BCR and regulatory coreceptors from Ramos cells and shows direct interaction in far-western lectin blots. **a** SILAC-based, mass-spectrometric screen of the BambL surface interactome on Ramos cells. BambL-biotin was allowed to bind to surface glycans only, and then the lectin-receptor complexes were extracted and identified my MS/MS peptide fingerprinting. The peptide hits enriched by BambL-biotin versus the negative control (BambL) of two experiments are plotted on a log_2_ fold-change scale. Peptides enriched ≥ fourfold were considered significant. Prominent hits are highlighted in the plot and listed in the table below (plot and table share the same color code). **b** Western blot validation of selected screening hits. Surface proteins were extracted with BambL-biotin as before. Whole-cell lysate (= pull-down input, ‘in’) and extract (‘ex’) were resolved by SDS-PAGE with different acrylamide concentrations (amount of loaded input ≙ 2% of extract amount). In control experiments, BambL-biotin was blocked with excess L-fucose. The sample succession in the images of CD45 and CD22 was originally swapped. These images were re-composed to maintain a consistent sample order across the presentation. **c** Far-western lectin blots identify direct binding partners of BambL. Pull-down input and extract were resolved by SDS-PAGE and transferred onto a membrane as before, then incubated with fluorescently labeled BambL (BambL-700). After washing, discrete bands were visible in the 700-nm channel (left image) which could be counter-stained in classical immunoblots in the next step (developed with fluorescent secondary antibodies in the 800-nm channel). Images look different because the staining and imaging procedure was individually optimized for each target. **d** Deglycosylated proteins no longer bind BambL. Whole-cell lysate was treated with the glycosidase PNGase F prior to the lectin blot to remove N-linked glycans. Glycosidase-treated proteins migrated farther (the µHC double band merged into a single band) and the lectin signal was almost absent from these sample lanes

Ramos B cells were cultivated in RPMI 1640 for stable-isotope labeling by amino acids in cell culture (SILAC RPMI 1640, Thermo Fisher) with 10% FCS for 14 days. For the first sample set, SILAC-labeled cells were incubated with 10 µg/mL BambL-biotin for 5 min on ice, and then washed to remove excess lectin. Unlabeled Ramos cells were incubated with non-biotinylated BambL and served as a negative control. For the second sample set, conditions were reversed (unlabeled cells were treated with BambL-biotin). After cell lysis in 50 mM Tris (pH 7.5), 150 mM NaCl, 1% (v/v) IGEPAL CA-630, 0.5% (w/v) sodium deoxycholate, BambL-receptor complexes were extracted using streptavidin-agarose beads (Thermo Fisher, 16 h rotating at 4 °C). The beads were washed and the two samples of each set were pooled. Proteins were eluted in Laemmli buffer and separated in SDS-PAGE. The gel was cut into slices, proteins therein were trypsinized and the resulting peptides purified with stop-and-go extraction (STAGE) tips (Thermo Fisher).

For mass-spectrometric analysis, samples were fractionated by nanoscale HPLC on a 1200 HPLC (Agilent) connected online to an LTQ Orbitrap XL mass spectrometer (Thermo Fisher). Fused silica HPLC-column tips with 75 μm inner diameter were self-packed with ReproSil-Pur 120 ODS-3 (Dr. Maisch) to a length of 20 cm. Samples were directly injected into the mass spectrometer as previously described [43]. The raw data files were processed with MaxQuant software [44]. Database searches were performed against a full-length human protein database containing common contaminants such as keratins and enzymes used for in-gel digestion. Carbamidomethyl-cysteine was set as fixed modification, and oxidation of methionine and protein amino-terminal acetylation were set as variable modifications. Triple SILAC was used as quantitation mode. The enzyme specificity was trypsin/P + DP with three allowed mis-cleavages. The MS/MS tolerance was set to 0.5 Da, and the mass precision of identified peptides after recalibration was in general less than 1 ppm. For identification and quantitation, the following settings were used: peptide and protein false-discovery rates were set to 0.01; maximum peptide posterior error probability was set to 0.1; minimum peptide length was set to 7; minimum number peptides for identification and quantitation of proteins was set to two, of which one must be unique; minimum ratio count was set to two; and only unmodified peptides and the variable modification were used for protein quantification. The “match between run” option was used with a time window of 2 min. We considered hits with more than a fourfold enrichment over the internal controls to be true interaction partners of BambL, resulting in a list of 120 proteins (online resource Tab. S1).

### Western blot analyses

Proteins were separated by SDS-PAGE and transferred onto a nitrocellulose membrane using semi-dry transfer following standard procedures. Homogenous protein transfer was checked with a reversible Ponceau S staining. All blocking and antibody incubation steps were carried out in TBS with 1% (v/v) Tween-20 and 3% (w/v) BSA unless mentioned otherwise.

Primary antibodies for western blots were purchased from Abcam (CD20: clone EP459Y #ab78237; CD21: clone EP3093 #ab75985; CD22: clone 2H1C4 #ab181771; CD45: clone MEM-28 #ab8216; IgM: clone EPR20731 #ab212201), Cell Signaling Technology (CD19: polyclonal, #3574; CD19 phospho-Tyr531: polyclonal #3571; Igα: clone D1X5C #13333; Igβ: clone D7V2F #96024; AKT: clone 40D4 #2920; AKT phospho-Ser473: clone D9E #4060; ERK1/2 p44/42 MAPK: clone 137F5 #4695; ERK1/2 phospho-Thr202/Tyr204: clone D13.14.4E #4370; SYK: polyclonal #2712; SYK phospho-Tyr525/526: clone C87C1 #2710), Bio-techne (galectin-9: polyclonal, #AF2045), Sigma-Aldrich (α-tubulin: clone B-5–1-2, #T5168), or Eurogentec (BambL: polyclonal, custom Ab against the BambL C-terminus).

Secondary antibodies for western blots were purchased either from Cell Signaling Technology (rabbit IgG HRP: #7076; mouse IgG HRP: #7074) and developed with Clarity Western ECL Substrate (Bio-Rad) or from Li-Cor (rabbit IgG IRDye 800CW: #926-32211, mouse IgG-IRDye 800CW: #926-32210) and imaged with an Odyssey CLx (Li-Cor).

### Western blot-based identification of BambL interaction partners on Ramos cells (Fig. 4b)

Ramos cells were collected by centrifugation, washed and incubated in cold PBS containing 5 µg/mL (180 nM) BambL-biotin for 30 min at 4 °C. Supplementing the solution with 1.6 mg/mL (10 mM) L-fucose 15 min prior to the experiment served as a negative control for carbohydrate-independent interactions of BambL. Cells were washed twice in cold PBS and lysed rotating for 90 min at 4 °C in pull-down lysis buffer: 50 mM Tris (pH 7.5), 150 mM NaCl, 1% (v/v) IGEPAL CA-630, 5% (v/v) glycerol, 1 mM EDTA, and protease inhibitors. The lysate was cleared by centrifugation and an aliquot of the supernatant was set aside to serve as input control (‘in’) during the analysis. From the remaining supernatant, BambL-receptor complexes were extracted with streptavidin-agarose beads (Thermo Fisher) for 4 h, rotating at 4 °C. After washing, proteins were eluted in 2 × Laemmli buffer, yielding the pull-down extract (‘ex’). Pull-down input and extract were separated side-by-side by SDS-PAGE with acrylamide concentrations ranging from 5 to 16% to resolve different protein sizes. The protein amount of input loaded corresponds to 2% of the extract amount. Western blots were then developed using standard procedures.

### Far-western lectin blots to identify direct BambL binding (Fig. 4c)

BambL-receptor complexes were extracted, separated by SDS-PAGE (different acrylamide densities for different targets), and transferred onto nitrocellulose as before. After transfer, the membrane was blocked in Intercept Protein-Free Blocking Buffer (Li-Cor), and then incubated shaking in a 25 µg/mL (0.9 µM) solution of BambL-700 in the same buffer for 1 h at RT. The membrane was washed extensively and imaged with the Odyssey CLx in both channels. The membrane was then processed in immunoblots as before, using fluorescently labeled secondary antibodies and recording their bands in the 800-nm channel. In addition, a second recording of the 700-nm channel was taken. By comparing the pictures before and after antibody staining, we eliminated the possibility of false-positive hits which could have been introduced by BambL binding to glycans of the detection antibodies.

### Enzymatic removal of N-linked protein glycans (Fig. 4d)

The endoglycosidase PNGase F cleaves Asn-linked mannose-, hybrid- and complex-type glycans at their core sugar unless their core is α(1 → 3) fucosylated. The enzyme tolerates core α(1 → 6) fucosylation. Ramos whole-cell lysates were prepared in pull-down lysis buffer as before, aiming for a total protein concentration of 1 mg/mL. Following the manufacturer’s recommendations (Sigma-Aldrich), the lysate was mixed with denaturing solution and heated (95 °C for 10 min), then split into two equal samples. One sample was treated with 500 units/mL PNGase F solution, shaking for 3 h at 37 °C. The reaction was stopped by heating the sample with Laemmli buffer. The other sample served as a treatment control. Both samples were assessed side by side in lectin blots as before.

### Western blot-based analysis of BCR signaling in Ramos cells (Fig. 5b)

**Fig. 5:**
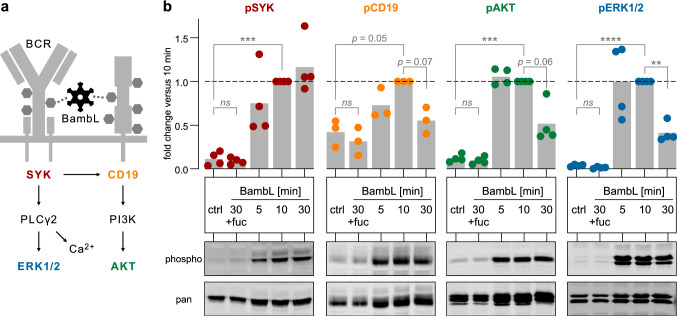
BambL induces BCR signaling. **a** Simplified scheme of intracellular BCR signaling. The protein phosphorylation of colored pathway members was used as a readout of their activation state (same color code applies to (**b**). **b** BambL induces phosphorylation of SYK, CD19, AKT and ERK1/2. Ramos cells were exposed to BambL for 5, 10 or 30 min, lysed and analyzed in western blots. As controls, cells were either left unstimulated (‘ctrl’) or supplemented with excess L-fucose and stimulated for 30 min (‘30 + fuc’). Phosphorylation-specific signal intensities (‘phospho’) were first normalized to their respective total proteins (‘pan’), then to their 10‑min sample (internal reference per experiment). Bars are mean fold changes (n = 3 for pCD19; n = 4 for pSYK, pAKT, pERK). Statistical significance (2-way ANOVA with Dunnett’s correction): ns (*p* > 0.05), ** (*p* ≤ 0.01), *** (*p* ≤ 0.001), **** (*p* ≤ 0.0001)

Ramos cells were seeded in complete medium and supplemented with 2 µg/mL BambL at different time points to obtain stimulation durations of 2, 10 and 30 min at 37 °C. The lectin concentration was chosen high to yield a robust phosphorylation readout. As negative controls, cells were either left unstimulated (‘ctrl’) or stimulated with BambL plus 10 mM L-fucose (‘30 + fuc’) for 30 min. Samples were then mixed with twice their volume of ice-cold PBS, collected by centrifugation, separated from their supernatant and lysed in M-PER lysis buffer (Thermo Fisher) with protease and phosphatase inhibitors for 20 min. The lysate was cleared by centrifugation and heated with Orange G loading buffer (Li-Cor). SDS-PAGE and western blotting were performed as before, using antibodies against SYK, CD19, AKT and ERK1/2. Total protein (‘pan’) and phosphorylated protein (‘phospho’) of each target were assessed on individual membranes. For quantification, the densitometric ‘phospho’ signal was normalized to its corresponding ‘pan’ signal, and then all lanes within one experiment were normalized to their 10-min sample (serving as a reliable internal reference). These relative values of individual experiments were used to calculate a mean fold change. Statistical significance of 10 min versus unstimulated was evaluated by 2-way ANOVA with Dunnett’s correction.

### Fluorescence microscopy of BambL internalization in Ramos cells (Fig. 6a)

**Fig. 6:**
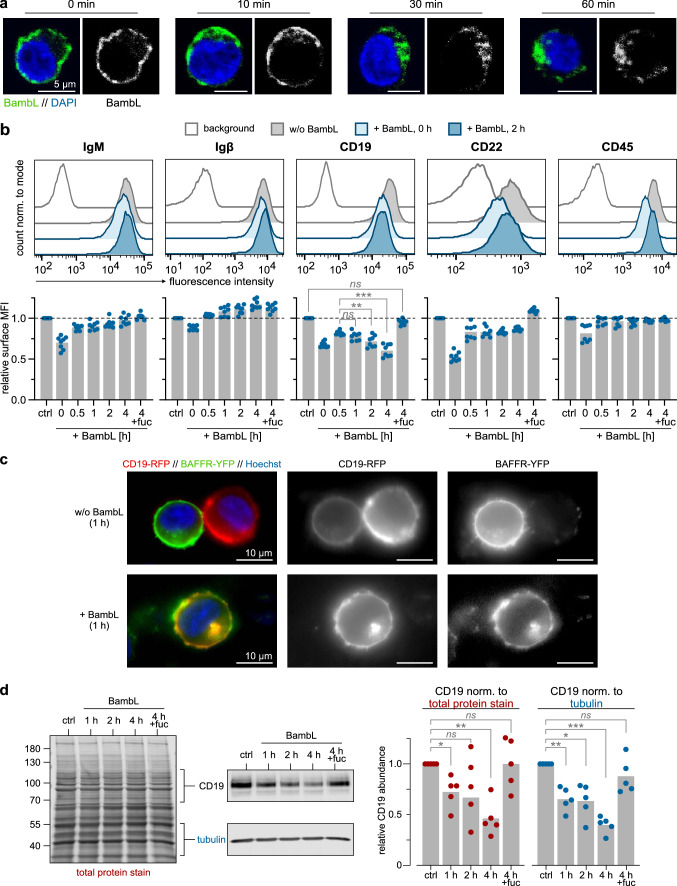
BambL is internalized and depletes surface and total CD19. **a** B cells internalize BambL. Ramos cells were loaded with BambL-488 (green) on ice, washed and transferred to warm medium to reinitiate internalization processes. Cells were fixed after indicated time points, counter-stained with DAPI (blue) and mounted for confocal microscopy. Representative confocal sections demonstrate a homogenous lectin distribution on the cell surface at time point 0 min, followed by an internalization wave and accumulation near the nucleus within 60 min. Scale bars: 5 µm. **b** BambL depletes surface CD19. Ramos cells were loaded with BambL on ice, washed and incubated in warm medium for indicated durations until flow cytometric analysis of surface proteins. As controls, cells were left without lectin (‘ctrl’) or BambL was supplemented with excess L-fucose (‘4 h + fuc’). Cells for time point “0 h” were loaded with lectin, washed and then analyzed without incubation in warm medium. Representative fluorescence histograms of four conditions are presented in the upper row. Bars below are mean fold changes of geometric mean fluorescence intensities (MFI) relative to ‘ctrl’ samples (*n* = 8). Statistical significance (2-way ANOVA with Dunnett’s correction): ns (*p* > 0.05), ** (*p* ≤ 0.01), *** (*p* ≤ 0.001). **c** B cells internalize CD19 upon BambL stimulation. Transfected BJAB cells expressing CD19-RFP and BAFFR-YFP were stimulated with BambL for 1 h (controls were left unstimulated), then imaged with a widefield microscope. Representative images demonstrate the internalization of both CD19 and BAFFR. **d** BambL induces degradation of internalized CD19. Ramos cells were incubated with BambL for indicated durations and analyzed in western blots. To control for differences in sample loading, the CD19 band intensities were normalized once to their total protein signal per lane (left image), once to tubulin. Bars are mean fold changes relative to unstimulated controls (*n* = 5). Either normalization strategy yielded a significant loss of total CD19 abundance over time. Statistical significance (2-way ANOVA with Dunnett’s correction): ns (*p* > 0.05), * (*p* ≤ 0.05), ** (*p* ≤ 0.01), *** (*p* ≤ 0.001)

Cold Ramos cells were loaded with BambL-488 on ice (2 µg/mL BambL in PBS, 5 min), washed and resuspended in warm complete medium to reinitiate internalization processes. After 0, 10, 30 or 60 min at 37 °C, cells were collected by centrifugation and fixed in 4% formaldehyde. After washing, nuclei were stained with DAPI, and cells were transferred to glass-bottom dishes in PBS with 20% glycerol. The cells were allowed to settle down and imaged with an Eclipse Ti-E A1R confocal microscope (Nikon), equipped with a 60 × oil-immersion lens with a numerical aperture of 1.49. The presented images are confocal slices of representative single cells.

### Receptor surface depletion and degradation in Ramos cells (Fig. 6b, d)

Each experiment comprised a 4-h time course of BambL-treated Ramos cells and controls, which were kept in complete medium in a cell culture incubator. For lectin treatment, the cells of each sample were first transferred to another plate, collected by centrifugation and washed once in cold PBS. The cells were resuspended in cold FACS buffer containing 4 µg/mL (145 nM) BambL and kept at 4 °C for 10 min to allow the lectin to bind to its surface targets, then collected by centrifugation and washed twice with PBS to remove unbound lectin. The cells were then resuspended in warm complete medium and transferred back to the original plate in the cell incubator which marked the start of the sample’s treatment duration. Treatments were started such that all conditions of one experiment were finished at the same time after 4 h. For example, the 2-h sample was left in warm medium for 1.5 h, loaded with BambL in cold buffer (0.5 h incl. washing steps), and then incubated in warm medium for the indicated treatment time of 2 h. As controls, cells were either (a) left untreated for 4 h (‘ctrl’), (b) left untreated for 3.5 h, loaded with BambL, but not moved back into warm medium (‘0 h’), or (c) loaded with BambL + 10 mM L-fucose and incubated in warm medium for 4 h (‘4 h + fuc’). When finished, the whole set of samples was prepared for flow cytometric analysis of surface markers following standard procedures and including a brief DAPI staining step to identify non-viable cells. Within the viable cell populations, geometric mean fluorescence intensities were corrected for background fluorescence using untreated and unstained Ramos cells, then normalized to the ‘ctrl’ sample which served as internal reference. Mean intensities were calculated from 8 independent experiments (error bars are SD). Statistical significance was evaluated by 2-way ANOVA with Dunnett’s correction.

To compare total CD19 abundance, cells were incubated with 1 µg/mL BambL in complete medium for up to 4 h, and whole-cell lysates were assessed in western blots as before. The signal intensities of CD19 bands were normalized twice: first to either a total protein stain per lane (Revert 700 Total Protein Stain, Li-cor) or α-tubulin serving as loading controls, then to the CD19 band intensity of the unstimulated sample within each experiment (internal reference). Using these relative values, mean fold changes of total CD19 abundance were calculated from 5 individual experiments (error bars are SD). Statistical significance was evaluated by 2-way ANOVA with Dunnett’s correction.

### Fluorescence microscopy of receptor internalization in BJAB cells (Fig. 6c)

BJAB cells stably expressing the fluorescent fusion proteins CD19-RFP and BAFFR-YFP were stimulated with 2.5 µg/mL BambL in FluoroBrite medium (Thermo Fisher), supplemented with Hoechst 33342 to stain nuclei, in glass-bottom culture dishes. Controls were incubated in medium without BambL. After 1 h, samples were imaged with an Axio Observer widefield microscope (Zeiss), equipped with a 63 × objective. Images show representative single cells.

### Additional software

Flow cytometry: FlowJo ver. 10 (Beckton Dickinson). Western blots: Image Studio ver. 5 (Li-Cor) and Fiji ImageJ ver. 1.52p [45]. Confocal microscopy: NIS Elements software ver. 5.20.01 (Nikon). Statistical analysis: Prism ver. 8 (GraphPad). Protein structure rendering: Open-source PyMOL ver. 1.8 [46]. Figure assembly and illustration: CorelDRAW ver. 2019 (Corel).

## Results

### BambL activates human peripheral B cells but becomes toxic at increasing concentrations

In a pilot experiment, we purified and cultivated peripheral human B cells in the presence of three BambL concentrations for 4 days (0.1, 0.5 or 2.5 µg/mL, corresponding to 3.6, 17.8 or 90 nM of functional, trimeric BambL, respectively). After 16 h of exposure, we noticed a concentration-dependent cytotoxic effect of the lectin. The loss of viable cells was marked by an increased DAPI sensitivity as well as decreased cell numbers in the ‘lymphocytes’ scattering gate (Fig. 2a, see also: online resource Fig. S1 for all 4 days). However, the lowest BambL concentration was well tolerated and successfully activated B cells as seen by their increased surface expression of CD69 (Fig. 2b). We also observed a stimulatory effect for 0.5 µg/mL BambL after 16 h, but the lectin’s toxicity dominated thereafter. Among the three subsets naive (IgD^+^, CD27^−^), marginal-zone (IgD^+^, CD27^+^) and class-switched memory B cells (IgD^−^, CD27^+^), the naive cells displayed the strongest relative increase in CD69 expression with an approximate doubling after 16 h treatment with 0.1 µg/mL BambL (Fig. 2c).

Whether or not B cell superantigens hold mitogenic potential has been discussed controversially in the literature. We followed up on this aspect for BambL in a cell proliferation assay: isolated B cells were labeled with a non-toxic, fluorescent dye whose concentration is cut by half with every cell division, and cultivated in the presence of 0.1 µg/mL BambL for 4 days. The proportion of viable cells decreased like in the unstimulated control and no changes in the fluorescence histogram of the proliferation dye were observed (Fig. S2). In the same time, the control stimulus produced 6 to 7 additional peaks in the histogram, representing the same number of cell division cycles. Therefore, BambL does not induce B cell proliferation ex vivo when used at a non-toxic yet activating concentration.

B cell superantigens induce caspase-dependent apoptosis. To assess the type of cell death upon BambL exposure, we isolated, stimulated and analyzed B cells using a combined annexin V and DAPI staining as a rough classification of apoptotic stages (Fig. S3). After 16 h exposure to 0.1 µg/mL BambL, the proportion of viable cells (annexin V^−^ DAPI^−^) was mildly decreased in comparison to the controls. However, at 1 µg/mL BambL we found about one third of the cells to be in a late apoptotic stage (annexin V^+^ and DAPI^+^) and about half as dead (annexin V^−^ DAPI^+^). Supplementing the stimulation medium with BAFF had only a marginal effect on the proportion of dead cells. These preliminary insights seem to agree with the mix of apoptosis and necrosis we had observed in murine B cells before [25], but more sophisticated analyses are required to accurately describe BambL-induced cell death pathways.

### BambL stimulates the expression of classical activation markers through PI3K-, SYK- and ERK1/2-dependent pathways

Continuing at 0.1 µg/mL BambL and with cell samples from multiple donors, we found the lectin to reliably induce the upregulation of CD69 and two more activation markers, the adhesion factor CD54 (ICAM-1) and the T cell stimulator CD86 (Fig. 3a). In contrast, the surface staining of CD80, which works in tandem with CD86, was unaffected. Among the subsets, we again observed the strongest response in naive B cells. In these experiments, we also included a F(ab’)_2_ antibody fragment against human IgM (‘anti-IgM’) at 1 µg/mL (9 nM) as a positive stimulation control.

Next, we sought to identify cellular pathways responsible for B cell activation after BambL stimulation. Purified B cells were pre-treated with inhibitors overnight, then exposed to BambL or anti-IgM and analyzed for their surface CD69 expression as before. Cells without inhibitor treatment served as controls. Inhibition of phosphoinositide 3-kinase (PI3K), spleen tyrosine kinase (SYK) or extracellular signal-regulated kinases (ERK1/2) each diminished B cell activation by either stimulant (Fig. 3b).

In addition to inhibiting cell activation intracellularly, we tested whether extracellular addition of fucose could block BambL binding and activity. This control served two purposes: first, to eliminate the possibility of BambL being recognized as a cognate antigen by the B cells, and second, to verify the absence of stimulatory contaminants like lipopolysaccharides (LPS) from our recombinantly produced lectin. Indeed, supplementing the stimulation media with excess fucose prevented cell death and activation by BambL but not anti-IgM (Fig. 3c). This result supports B cell activation by BambL to be purely glycan-driven.

Together, our results demonstrate that a sub-toxic dose of BambL effectively activates human B cells in a glycan-dependent fashion. Further, our data suggest BambL signals through the BCR, which matches our earlier study with murine B cells [25]. In mouse, we had found BambL injection to deplete B cells but not T cells, which suggests different cellular responses to the lectin [25]. Furthermore, influenza HA had been demonstrated to activate peripheral B cells but not T cells in vitro in another study [24]. Therefore, we tested whether human T cells could be activated by BambL, and found both CD4^+^ and CD4^−^ T cells to upregulate CD69 after 16 h BambL exposure (online resource Fig. S4a). However, the relative increases versus unstimulated control samples were significantly less than that of B cells in the same samples. In a follow-up experiment, we noted the downmodulation of the checkpoint regulator TIGIT (T cell immunoreceptor with immunoglobulin and ITIM domain, [47, 48]) but upregulation of CD25 (IL-2 receptor alpha chain) among CTLA4 (cytotoxic T lymphocyte-associated protein 4)-expressing CD4^+^ T cells (Fig. S4b). Cytotoxic T lymphocytes (CD8^+^ CD56^−^) also experienced an increase in the CD25^+^ CTLA4^+^ fraction, but no changes in TIGIT expression (Fig. S4c). Our data suggest BambL induces a regulatory T cell phenotype (T_reg_), unlike anti-CD3 stimulation. However, more extensive work is required to accurately characterize T cell responses to BambL.

### BambL binds to the BCR and regulatory coreceptors on Ramos B cells

Our observations had us speculate about the molecular targets of BambL. Fucose has previously been identified within glycans of secreted human immunoglobulins, but the precise glycosylation patterns of membrane-bound Igs are unknown. Therefore, we investigated the B cell surface interactome of BambL with a heavy-isotope labeling and mass-spectrometric approach (SILAC) using the Ramos B cell line. Cells were loaded with biotinylated lectin (BambL-biotin) in the cold to minimize internalization and allow for binding of surface glycans only. Lectin-receptor complexes were then extracted, digested and identified by their peptide mass fingerprints. Cells treated with non-biotinylated BambL served as negative controls. The mass-spectrometric protein hits enriched in the BambL-biotin-treated samples of two experiments are mapped in Fig. 4a. Since fucose is a common component of protein glycans, we obtained a broad spectrum of interaction candidates. Here, we identified the BCR complex, namely µHC and Igβ (or CD79b), and its regulatory coreceptors CD19, CD22 (Siglec-2) and CD45 (B220, PTPRC). Adhesion factors and MHC class 2 members were represented among the top hits, too. We further identified the death receptor FAS-R (CD95, APO-1) and the metabolically important insulin receptor among the enriched proteins. Protein hits of particular interest to our study are summarized in Fig. 4a (for a full list, see online resource Table S1).

To validate potential B cell targets from the interactome screen, biotinylated BambL was used as bait for further pull-down and western blot analyses (Fig. 4b). BambL-biotin extracted the mature, fully glycosylated µHC as it is present on the cell surface but not the premature protein of lower molecular weight [49, 50]. Similarly, higher Igβ bands were more pronounced in the extract than lower bands. However, we were unable to obtain a robust and reproducible result for Igα. The BCR regulators CD19, CD22 and CD45 produced strong signals, and CD19 was complemented by its membrane complex partner CD21 (CR2) which had not been identified in the screen before.

In addition, we detected CD20, whose expression has recently been found to be intimately involved in the organization of these surface receptors [51]. We further noticed the extraction of galectin-9, a galactoside-specific lectin recently described to negatively regulate BCR activity through CD45 binding and activation of the CD22-LYN-SHP-1 pathway [52]. Importantly, all proteins reported here were absent from the control extracts in which BambL had been blocked with excess fucose.

We wondered whether the lectin’s interaction with the aforementioned receptors was direct or indirect. To answer this question, we established a far-western lectin blot assay similar to Thuenauer and colleagues [32, 53], instead utilizing infrared fluorescence dyes. Briefly, Ramos lysates and pull-down extracts were resolved by SDS-PAGE and transferred onto a nitrocellulose membrane, then incubated in a solution of fluorescently labeled BambL (BambL-700). After washing, we obtained several intense bands in input and extract lanes (Fig. 4c, image on the left). The membrane was then probed with antibodies in classical immunoblots using fluorescent secondary antibodies and scanned for signal overlaps (images on the right). The staining intensities of different membrane segments required us to optimize conditions for each target individually, which explains why these images look different than the full blot on the left. We found the bands of µHC, CD19, CD22 and CD45 to overlap well with prominent lectin bands.

For µHC, BambL-700 almost exclusively stained the upper band which agrees with the previous extraction results showing minimal interaction with the premature µHC. Similarly, the lectin blot produced a strong overlap in the upper range of Igβ. For CD21, the receptor signal overlaps with a thin lectin-marked band but their shapes are very different. It is possible that only a fraction of CD21 in the extract carries fucose. Alternatively, BambL-700 may be binding to another, unidentified protein of similar size.

To exclude possible glycan-independent BambL binding in the lectin blots, Ramos lysates were treated with PNGase F prior to SDS-PAGE. The amidase cleaves most N-linked glycans at their core between asparagine and the first N-acetylglucosamine [54]. Fucose, which is commonly incorporated in N glycans with an α(1 → 6) glycosidic link to the innermost N-acetylglucosamine, is hereby removed as well. Consequently, minimal BambL-700 staining was obtained in samples treated with PNGase F (Fig. 4d). As expected, the deglycosylated proteins migrated faster and the previously observed µHC double band was merged into a single band.

Collectively, the pull-down assay and lectin blots suggest the BCR components µHC and Igβ as well as coreceptors CD19, CD22 and CD45 to carry fucosylated glycans and to constitute direct targets for BambL on the surface of Ramos B cells.

### BambL induces BCR signaling

We then wondered whether the direct interaction between BambL and BCR stimulated the intracellular BCR signaling pathway. Therefore, we analyzed the phosphorylation of downstream signaling proteins (Fig. 5a) in BambL-treated Ramos cells in western blots. Indeed, BambL readily activated BCR signaling within minutes of stimulation (Fig. 5b). We observed phosphorylation of the early kinase SYK, and further downstream, of the CD19 cytoplasmic tail, AKT and ERK1/2. Interestingly, while the phosphorylation levels of CD19, AKT and ERK1/2 peaked at around 10 min and then dropped (consistent with inhibitory BCR feedback loops), SYK phosphorylation remained high throughout the experiment. Blocking the lectin with fucose prevented phosphorylation of all targets.

### BambL-stimulated B cells internalize and degrade CD19

Normal BCR engagement by cognate antigens triggers the internalization of the receptor–antigen complex. However, BambL does not bind the same BCR regions as antigens do. We, therefore, asked whether BambL, too, would induce BCR internalization. In a pilot experiment, Ramos cells were loaded with fluorescently labeled BambL-488 on ice, excess lectin was washed out, and cells were moved into warm medium to reinitiate the internalization machinery. Samples were fixed after different time points, counter-stained with DAPI and analyzed with confocal microscopy (Fig. 6a). At time point ‘0 min’ directly after the washout, BambL-488 almost homogenously covered the cell surface. With increasing time in warm conditions, however, the lectin first clustered on one side of the cell and was then internalized. After 60 min, the majority of lectin had been internalized. This provided an insight into the kinetics of potential receptor internalization.

Next, we assessed the surface levels of IgM, Igβ, CD19, CD22 and CD45 after different time points of BambL stimulation with flow cytometry (Fig. 6b). Again, Ramos cells were loaded with BambL on ice and excess lectin was washed out before cultivating the cells in warm medium for up to 4 h. In samples that were analyzed directly after the washing step and before incubation in warm medium (‘0 h’), all five receptors of interest experienced a marked loss of signal compared to the lectin-free samples (‘ctrl’). We do not interpret this drop to stem from receptor internalization because the cold temperature suppressed endocytosis during all steps. Instead, we suggest that BambL can partly mask the epitopes of the staining antibodies. We found the initial loss of fluorescence to recover over time, probably due to lectin dissociation and cellular export of new receptors. CD19, however, presented a notable exception: after partial recovery within 0.5 h in warm medium, the CD19 signal consistently decreased. Since unbound lectin had been washed out, this loss of signal represented genuine receptor depletion. Supplementing the lectin solution with fucose (‘4 h + fuc’) prevented CD19 depletion. When we replaced BambL with anti-IgM in the same experimental setup, CD19 levels remained constant (online resource Fig. S5).

We asked whether the depletion of surface CD19 could be explained by receptor internalization. To test our hypothesis, BJAB cells (another human B cell line) were co-transfected with two fusion proteins: CD19 fused to red fluorescent protein (CD19-RFP), and B cell-activating factor receptor fused to green fluorescent protein (BAFFR-YFP). We imaged cells with confocal microscopy and found both conjugates successfully expressed on the cell surface (Fig. 6c). After 1 h of BambL exposure, however, cells had substantially internalized CD19-RFP and BAFFR-YFP, whose intracellular signals largely colocalized near the nucleus.

Next, we analyzed whole-cell lysates of BambL-treated Ramos cells in western blots to assess total CD19 protein levels (Fig. 6d). To correct for loading differences, we compared two normalization controls: (a) a total protein stain, whose intensity is rather insensitive to changes in the abundance of single proteins, and (b) tubulin. Using either loading control, we measured a significant decrease in CD19 protein levels after 1 h of BambL stimulation. Again, fucose supplementation prevented the effects of BambL incubation.

Together, these experiments demonstrate a striking difference between BCR engagement by BambL or anti-IgM. Both stimuli bind the constant Ig domain and both induce intracellular BCR signaling. But unlike anti-IgM, BambL does not persistently cause BCR internalization which is a prerequisite for intracellular processing of a cognate antigen. Instead, the lectin triggers the internalization and degradation of the important coreceptor CD19. The other two receptors we monitored here, CD22 and CD45, were not depleted by BambL. However, our microscopy analysis of BAFFR-YFP, a receptor which was not among the interaction candidates of the mass-spectrometric screen, suggests that more surface proteins may be subject to internalization.

## Discussion

### BambL binds to the BCR and coreceptors on human B cells

We identified µHC and Igβ of the BCR complex as BambL interaction partners on the surface of Ramos cells, along with four prominent regulators of BCR signaling: CD19, CD21, CD22 and CD45. All six proteins were well detectable in western blots of the pull-down extract. Moreover, the lectin blots yielded good signal overlaps of lectin and antibodies against µHC, Igβ, CD19, CD22 and CD45. It is within the technical limitations of the lectin blot assay that BambL-700 may bind another fucosylated protein at the same migration height as our receptor of interest. In addition, some glycans stained here may not be accessible under physiological conditions due to native protein folding and localization. Nevertheless, our results strongly suggest the BCR, CD19, CD22 and CD45 to constitute direct targets of BambL on human B cells.

Ramos cells predominantly express IgM-type BCRs [55, 56]. Surprisingly, we have previously found IgM to be dispensable for B cell activation by BambL in a murine knockout line [25]. We hypothesize that different µHC glycan compositions in human and murine B cells underlie this disagreement. Several studies have identified fucose residues in secreted IgM of both mice [57–60] and humans [61–64]. However, the glycans of secreted Igs may differ from those of membrane-anchored BCR-Igs, and the cancer models typically employed in such studies may have acquired abnormal glycosylation patterns. Moreover, glycosylation patterns can change during cell maturation, as has been demonstrated for the IgM-BCR during B cell development [50], and reflect a cell’s microenvironment [65]. Species-dependent differences further exacerbate the comparability of studies.

### BambL triggers BCR signaling

BambL stimulated downstream BCR signaling within minutes in Ramos cells. In addition, our kinase inhibition screen with peripheral B cells identified SYK, PI3K and ERK1/2 as essential components of the cell activation mechanism. These data agree with our earlier study in the murine system in which knockout of either IgD-BCR, CD19 or SYK prevented B cell activation by BambL [25]. Together, these results point toward canonical BCR signaling upon BambL engagement, but more work is needed to further probe this question.

BCR binding by antigens translates to intracellular signaling when the antigens establish multivalent interactions and disperse BCR nano-clusters [66–68] or link isolated BCRs [69, 70]. This receptor reorganization renders the intracellular Igα/β ITAMS available for phosphorylation. Similarly, we propose that BambL crosslinks multiple receptors and acts as a hexavalent clustering hub on B cell membranes. Support for this model comes from previous reports of receptor clustering by multivalent lectins [30, 32, 71–73], particularly by the fucose-binding *Ralstonia solanacearum* lectin RSL which shares remarkable structural homology with BambL [74, 75]. We propose it is this receptor crosslinking by which BambL modulates naive BCR organization of resting B cells and triggers intracellular downstream signaling pathways.

CD19, CD22, and CD45, which we identified as additional BambL-binding targets, classically regulate these pathways. CD19 and its membrane complex partners CD21 and CD81 amplify BCR signaling by linking it to the complement system [76]. Hence, we presume that BambL binding to CD19 promotes B cell activation, particularly should CD19 become crosslinked to the BCR. CD22, on the other hand, normally produces a negative feedback loop for BCR signaling: CD22 activates the phosphatase SHP-1, which in turn counteracts stimulatory kinases [77]. However, on resting B cells, CD22 largely resides in homomultimeric, inactive clusters [78]. BambL may crosslink and stabilize these CD22 clusters which prevents the individual receptors from counteracting BCR activation. Finally, CD45 holds an ambivalent part as it can both stimulate and inhibit BCR signaling [79, 80]. However, extensive CD45 ligation has been shown to induce apoptosis in B and T cells [81] which may fit our observation of cell death after BambL stimulation.

In addition to establishing new links between surface proteins, we propose that BambL also challenges glycan-mediated interactions naturally prevailing in the B cell membrane. The competitors need not even share the same sugar specificity, because BambL may cloak nearby target sugars of other lectins. In doing so, hexavalent BambL with its strong avidity may outcompete other lectins like monovalent CD22 [82–84] or divalent galectin-9, which also coprecipitated in our pull-down experiments. On murine B cells, the galactose-specific lectin was demonstrated to associate the BCR with CD45 and CD22 and suppress BCR signaling by activating the LYN-CD22-SHP-1 pathway [52, 85, 86]. By competing for the same receptors, BambL may prevent galectin-9 from fulfilling this inhibitory function. The list of diverse binding candidates we obtained in our mass-spectrometric screen emphasizes that BambL’s interactions on B cells may be manifold and their implications complex.

### BambL depletes CD19 but not IgM

Using two human B cell lines, we demonstrated that BambL stimulates the internalization and degradation of CD19, the physiological consequences of which we can only speculate about at this point. Potentially, the loss of CD19 modulates the stimulation threshold of BCR activation and turns B cells less responsive to bacterial antigens. Interestingly, CD19 downregulation has also been reported to occur upon stimulation with the B cell superantigen SpA [15]. We did not, however, observe changes in the surface levels of the BCR on BambL-treated Ramos cells. It is tempting to infer that B cells cannot process BambL for MHC presentation like antigens. Future studies may answer the question whether B cells can generate an antibody response to BCR-binding lectins.

In contrast to BambL, the IgM-crosslinking antibody we used as a control stimulus induced strong BCR surface depletion but did not modulate CD19 presence. One explanation for this difference is that binding of BambL or anti-IgM are sufficiently distinct on the molecular level to trigger different cellular responses. Alternatively, the reason may lie beyond BCR ligation alone and result from the lectin’s other surface interactions.

### *BambL pan*-*activates human blood B cells and stimulates the expression of classical activation markers*

Purified B cells were sensitive to small amounts of BambL, with 0.1 µg/mL (3.6 nM) being the lowest concentration we tested. Within 16 h, BambL stimulated the upregulation of activation markers CD69 and CD54, which aid in retaining B cells in lymphatic tissue and establishing the immunological synapse with T-helper cells [87–89]. We further detected the upregulation of CD86, which provides costimulatory signals for T cell activation via its interactions with CD28 on T cells and contributes to B cell differentiation [90–92]. Importantly, saturating BambL with excess fucose completely abolished B cell activation and cell death in all our assays. Thus, the stimulation mechanism is lectin-driven, glycan-mediated and independent of bacterial pattern recognition machineries like toll-like receptors. Our results demonstrate that BambL elicits a polyclonal activation independent of BCR specificity, which may reduce the mobility of B cells and prime them for T cell engagement.

Our results excellently match a previous report of B cell activation by the trimeric, trivalent and sialic acid-specific influenza hemagglutinin (HA, cf. Fig. 1) [24]. Sialic acid, like fucose, is a common component of protein and lipid glycans. Human B cells responded to 50 nM HA with the upregulation of surface CD69 and CD86, but not CD80. By reconstituting different IgM sequences with or without N-glycosylation sites in a Ramos model, the authors also elegantly demonstrated the sugar-dependent mode of action and concluded that the lectin could pan-activate B cells. Interestingly, an earlier publication from another group had ascribed HA a mitogenic impact on isolated murine B cells ex vivo [93], but this effect was not confirmed in the later study. The pentameric and pentavalent cholera toxin B subunit (CtxB), which also engages sialic acid, was reported to induce MHC-II and CD86 upregulation in murine B cells, accompanied by only a modest increase in CD69 and CD80 [94]. Notably, the CtxB concentration applied in that study was much higher (175 nM).

### Higher BambL concentrations become increasingly toxic to B cells

**Fig. 7:**
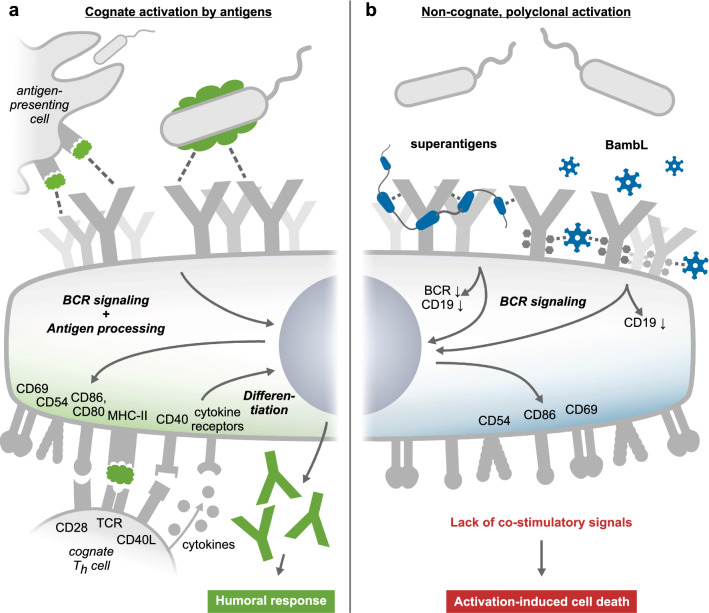
Proposed models of cognate vs. non-cognate and polyclonal B cell activation. **a** Classical cognate activation by antigens. Arrayed antigens (green) on the surface of a pathogen or an antigen-presenting cell stimulate BCR signaling, expression of activation markers CD69, CD86/80 and CD54, internalization of the BCR-antigen complex, and antigen processing for presentation on MHC-II. A complementary T helper cell (T_h_) provides costimulatory signals through CD40L and cytokines such as IL-2, which initiate a B cell differentiation program into antibody-secreting plasma cells. **b **Proposed model for non-cognate and polyclonal activation. B cell superantigens like SpA crosslink conserved BCR sites, downmodulate BCR and CD19 expression, and pan-activate B cells irrespective of their antigen specificity. We propose multivalent lectins like BambL achieve a similar effect by crosslinking BCR glycans. In the absence of sufficient survival and differentiation signals, the B cells succumb to exhaustion in a form of activation-induced cell death

While 0.1 µg/mL BambL activated peripheral B cells without an apparent cytotoxic effect, the loss of viable cells at higher lectin concentrations was substantial. Interestingly, the FAS receptor (CD95) was identified as a candidate target of BambL in the mass-spectrometric interactome screen. Its engagement could explain caspase-dependent cell death—however, we were unable to verify the interaction in western blots of the pull-down extract. Therefore, we hypothesize that upon high local BambL concentrations B cells succumb to the same FAS-independent fashion of activation-induced cell death (AICD) that has been postulated for B cell superantigens [1, 3, 8, 15, 16, 22]: excessive receptor engagement and polyclonal activation generates high cellular demands for nutrients and survival stimuli from cytokines and cognate T helper cells, the lack of which drives the B cells into exhaustion and apoptosis (cf. graphical summary in Fig. 7).

### BambL acts as a superantigen toward human B cells

In summary, our results demonstrate that by binding glycans of BCR-Igs and other surface targets, BambL stimulates BCR signaling, CD19 depletion and the expression of classical activation markers. BambL also becomes increasingly cytotoxic through mechanisms that may be explained by AICD. Importantly, BambL pan-activates human B cells irrespective of their antigen specificity. Collectively, our proposed mode of action for BambL bears much functional similarity to classical B cell superantigens, with two major distinctions. First, the lectin does not bind amino acids but engages the BCR via heavy-chain glycans. Second, BambL is not exclusive for the BCR but may crosslink a multitude of receptors on the B cell surface.

BambL is produced by *Burkholderia ambifaria* [42], an opportunistic human pathogen and member of the *Burkholderia cepacia* complex (Bcc), which is accountable for severe acute and chronic lung infections [36, 38, 95–98]. The ‘cepacia syndrome’ caused by Bcc species includes necrotizing pneumonia, leukocytosis and fatal pulmonary deterioration. Like the closely related genus of *Pseudomonas*, *Burkholderia* species form resilient biofilms and are resistant to most antibiotics and antiseptic solutions [99, 100]. This resistance limits therapeutic options and increases their threat in hospital environments and to immuno-compromised individuals. Host epithelial barriers are a first line of defense, limiting bacterial colonization to exterior surfaces of the body. However, biofilms exert physical forces on their underlying substrate, deteriorate epithelial cell–cell junctions and can create entry points to underlying tissue [101]. To date, the natural stimulus for *B. ambifaria* to release BambL is unknown. When grown as a biofilm on agar plates, *B. ambifaria* constitutively produces BambL but retains the lectin in the cytosol [25]. Future work on the pathology of *Burkholderia* infections may not only shed light on when and how certain virulence factors are released, but also on the implications of massive non-cognate B cell activation for disease progression. So far, the reports of superantigens generally agree in that dysregulated polyclonal activation of B cells impedes the clearance of bacterial infections. However, an important question remains unanswered: Could it be possible for B cells to process low doses of BambL for antigen presentation and antibody production? It is tempting to speculate that, in the physiological context, the binding of cognate antigen to BCR-BambL complexes may induce receptor internalization and enable an adaptive humoral response to BambL after all. We are excited to see what insights future studies on carbohydrate-mediated B cell activation will bring to light (Fig. 7).

## Supplementary Information

Below is the link to the electronic supplementary material.

Supplementary file1 (DOCX 1834 KB)

## Data Availability

The datasets generated and analyzed during the current study are available from the corresponding authors upon reasonable request.

## Abbreviations

AICD: Activation-induced cell death
AKT: AKR mouse strain thymoma protein, or protein kinase B
anti-IgM: F(ab’)2 antibody fragment against human IgM
BAFF: B cell-activating factor
BambL: Fucose-binding *Burkholderia ambifaria* lectin
Bc2L-A: *Burkholderia cenocepacia* Lectin A
Bcc: *Burkholderia cepacia* Complex
BCR: B cell antigen receptor complex
CDR: Complementarity-determining region
CTLA4: Cytotoxic T lymphocyte-associated protein 4
CtxB: Cholera toxin B subunit
DAPI: 4′,6-Diamidino-2-phenylindole
ERK1/2: Extracellular signal-regulated kinases 1/2
FAS: Fas cell surface death receptor, a.k.a. CD95
HA: Influenza hemagglutinin
HLA: Human leukocyte antigen
Ig: Immunoglobulin
ITAM: Immunoreceptor tyrosine-based activation motif
LecB: Fucose-binding lectin of *Pseudomonas aeruginosa* (a.k.a. PA-IIL)
LPS: Lipopolysaccharides
LYN: LCK/YES-related novel tyrosine kinase
MFI: Geometric mean fluorescence intensity
MHC: Major histocompatibility complex
MS: Mass spectrometry
PBMC: Peripheral-blood mononuclear cell
PI3K: Phosphatidylinositide 3-kinase
PDB: RCSB protein data bank
PNGase F: Peptide:N glycosidase F
RSL: Fucose-binding *Ralstonia solanacearum* lectin
SHP-1: Src homology region 2 domain-containing phosphatase-1
SILAC: Stable isotope labeling by amino acids in cell culture
SpA: Staphylococcal protein A
SYK: Spleen tyrosine kinase
TIGIT: T cell immunoreceptor with Ig and ITIM domains.
ANOVA: Analysis of variance
BSA: Bovine serum albumin
DMSO: Dimethyl sulfoxide
EDTA: Ethylenediaminetetraacetic acid
FACS: Fluorescence-activated cell sorting
FCS: Fetal calf serum
FPLC: Fine pressure liquid chromatography
HABA: 4’-Hydroxyazobenzene-2-carboxylic acid
HPLC: High-pressure liquid chromatography
IMDM: Iscove’s modified Dulbecco’s medium
IPTG: Isopropyl β-D-1-thiogalactopyranoside
MACS: Magnetic cell sorting
MFI: Mean fluorescence intensity
MS: Mass spectrometry
PBS: Phosphate-buffered saline
RPMI: Roswell Park Memorial Institute medium
SDS-PAGE: Sodium dodecyl sulfate–polyacrylamide gel electrophoresis
TBS: Tris-buffered saline
Tris: Tris-(hydroxymethyl)-aminomethane
