# Supplementary material for: Bacterial lectin BambL acts as a B cell superantigen
Source: Cell Mol Life Sci. 2021 Nov 3;78(24):8165–86. doi: 10.1007/s00018-021-04009-z (PMC8629787; doi:10.1007/s00018-021-04009-z)
Supplement: Supplementary file 1 — Supplementary file1 (DOCX 1834 KB) [file 18_2021_4009_MOESM1_ESM.docx]

# Supplementary Material

Bacterial lectin BambL acts as a B-cell superantigen

Marco Frensch^1,2,3^, Christina Jäger^1,2^, Peter F. Müller^1,2^, Annamaria Tadić^1,2^, Isabel Wilhelm^1,2,4^, Sarah Wehrum^1,2^, Britta Diedrich^5,6^, Beate Fischer^7^, Ana Valeria Meléndez^1,2,4^, Joern Dengjel^5,6^, Hermann Eibel^7^* and Winfried Römer^1,2,3,4,8^*

## Affiliations

^1^ Faculty of Biology, University of Freiburg, Freiburg, Germany.

^2^ Signaling Research Centers BIOSS and CIBSS, University of Freiburg, Freiburg, Germany.

^3^ International Max Planck Research School for Molecular and Cellular Biology IMPRS-MCB, Max Planck Institute of Immunobiology and Epigenetics, Freiburg, Germany.

^4^ Spemann Graduate School of Biology and Medicine SGBM, University of Freiburg, Freiburg, Germany.

^5^ Department of Biology, University of Fribourg, Switzerland.

^6^ Department of Dermatology, University Medical Center and University of Freiburg, Freiburg, Germany.

^7^ Center for Chronic Immunodeficiency CCI and University Medical Center Freiburg, Freiburg, Germany.

^8^ Freiburg Institute for Advanced Studies (FRIAS), University of Freiburg, Freiburg, Germany.

* corresponding authors

## To whom correspondence should be addressed:

Prof. Dr. Winfried Römer, [winfried.roemer@bioss.uni-freiburg.de](mailto:winfried.roemer@bioss.uni-freiburg.de)

Prof. Dr. Hermann Eibel, [hermann.eibel@uniklinik-freiburg.de](mailto:hermann.eibel@uniklinik-freiburg.de)

## Supplementary Methods and Materials

### Flow cytometric analyses

After stimulation, live cells were transferred into cold FACS buffer (PBS, 5% FCS, 0.5 mM EDTA). Antibody staining was carried out according to the manufacturers’ recommendations, followed by a brief staining with DAPI to mark non-viable cells. Antibodies for flow cytometry were purchased from Biolegend (CD4: clone RPA‑T4, catalogue #300511, and clone OKT4, #317427; CD8a: clone RPA‑T8 #301009; CD19: clone HIB19 #302218 and #302233; CD22: clone HIB22 #302517; CD45: clone HI30 #304020; CD69: clone FN50 #310906; CD79b: clone CB3-1 #341404; IgM: clone MHM-88 #314506; TIGIT: clone A15153G #372723), BD Biosciences (CD3: clone HIT3a #561802; CD25: clone 2A3 #335807; CD56: clone B159 #557699; CTLA4: clone BNI3 #560939), eBioscience (CD8a: clone RPA‑T8 #45-0088-42), Invitrogen (CD8a: clone 3B5 #MHCD0805) or Southern Biotech (IgM: polyclonal #2022‑01).

### Cell proliferation assay (Fig. S2)

Purified B cells were labeled with CellTrace Violet according to the manufacturer’s recommendations (Thermo Fisher) and cultivated in presence of 0.1 µg/mL BambL for 4 days. As controls, cells were stimulated with 0.1 µM CpG oligonucleotides + 25 ng/mL BAFF (Thermo Fisher) or left unstimulated. Representative scattering plots and fluorescence histograms are shown for each condition (n = 4).

### Cell death analysis (Fig. S3)

Purified B cells were incubated with 0.1 µg/mL (3.6 nM) BambL, 1.0 µg/mL (36 nM) BambL, 1 µg/mL (9 nM) F(ab’)_2_ anti‑human IgM (‘anti‑IgM’, Southern Biotech), or left unstimulated for 16 h (n = 2). A second set of samples was supplemented with 25 ng/mL BAFF (Thermo Fisher). Cells were stained with Alexa Fluor 647-conjugated Annexin V (Southern Biotech) and DAPI.

### Comparison of B and T cell activation (Fig. S4a)

Total PBMCs were stimulated with either 0.1 µg/mL BambL, 1 µg/mL anti‑IgM, anti‑CD3 (surface coating with a self-made ‘UCHL1’ antibody: 1 mg/mL for 1 h prior to cell seeding), or left unstimulated for 16 h. Cells were stained with antibodies against CD19 or CD3 + CD4 (to identify B or T cells, respectively), and against CD69 (all samples). Frequencies of viable cells were calculated relative to unstimulated controls. Bars represent mean fold changes ± SD (n = 3). Statistical significance was evaluated by 2‑way ANOVA with Tukey’s correction.

### Analysis of T cell surface markers (Fig. S4b and c)

Total PBMCs were stimulated with 0.1 µg/mL BambL or 1 µg/mL anti‑CD3 (UCHL1) for 16 h. Control samples were left unstimulated. Cells were stained with antibodies against CD4, CD8, CD56, CD25, CTLA4 and TIGIT. Bars represent mean frequencies of viable cells ± SD (n = 4). Statistical significance was evaluated by 2‑way ANOVA with Tukey’s correction.


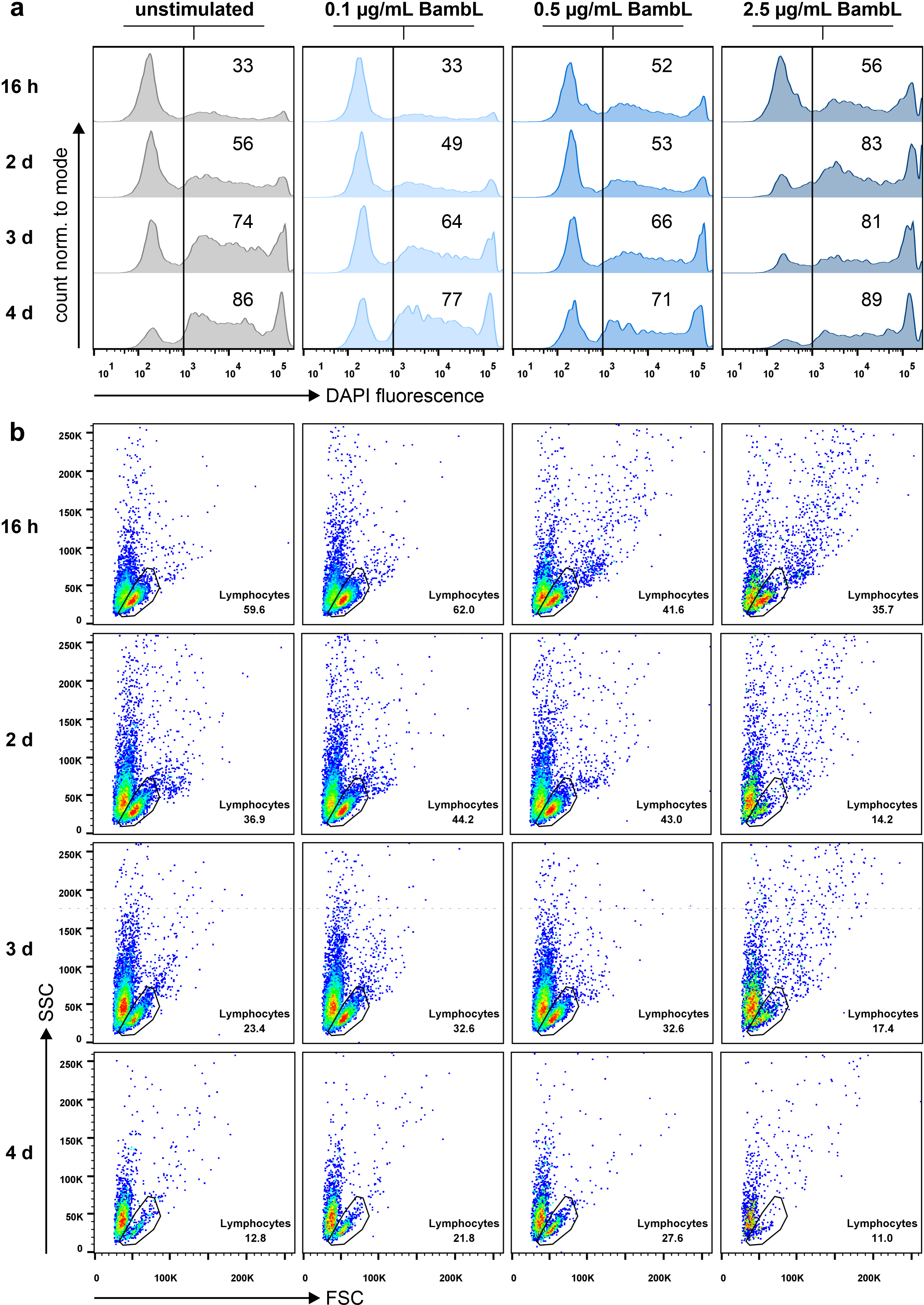


**Fig. S1 Time course of DAPI sensitivity and cell counts in Lymphocytes gate (supplement to Fig. 2)**

B cells were isolated from PBMCs and cultivated in presence of three different BambL concentrations or left unstimulated for 4 days (n = 1 for each condition and time point). Samples were analyzed by flow cytometry after each day.

**a** **DAPI sensitivity.** Histograms of DAPI fluorescence; numbers indicate the frequencies of cells considered DAPI-positive in each sample.

**b** **Light scattering behavior.** Sideward (SSC) versus forward (FSC) scattering; numbers indicate frequencies in the lymphocytes gates.


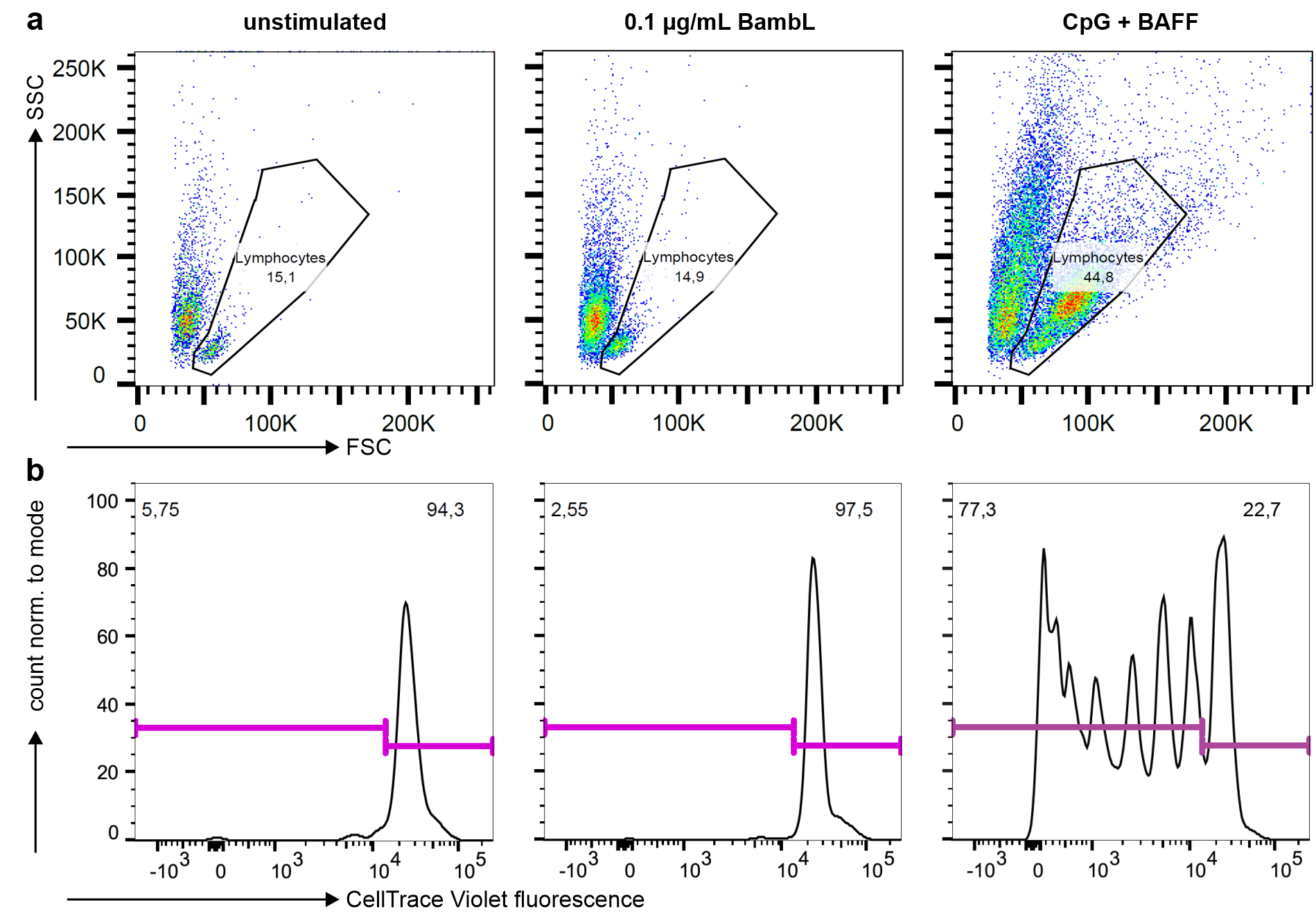


**Fig. S2 BambL does not induce B cell proliferation at a non-toxic concentration**

Purified B cells were labeled with CellTrace Violet and cultivated in presence of 0.1 µg/mL BambL for 4 days. As controls, cells were stimulated with CpG oligonucleotides + BAFF or left unstimulated. Plots are representative of n = 4.

**a Proportion of viable B cells is similar to unstimulated controls.** Light scattering plots demonstrate no changes in viable B cell numbers upon BambL stimulation, unlike CpG + BAFF.

**b Fluorescence histogram of the proliferation dye remains unchanged.** BambL-treated cells present a single fluorescence peak, whereas CpG + BAFF induced 6 to 7 additional peaks at lower fluorescence intensity (representing 6 to 7 cell divisions).


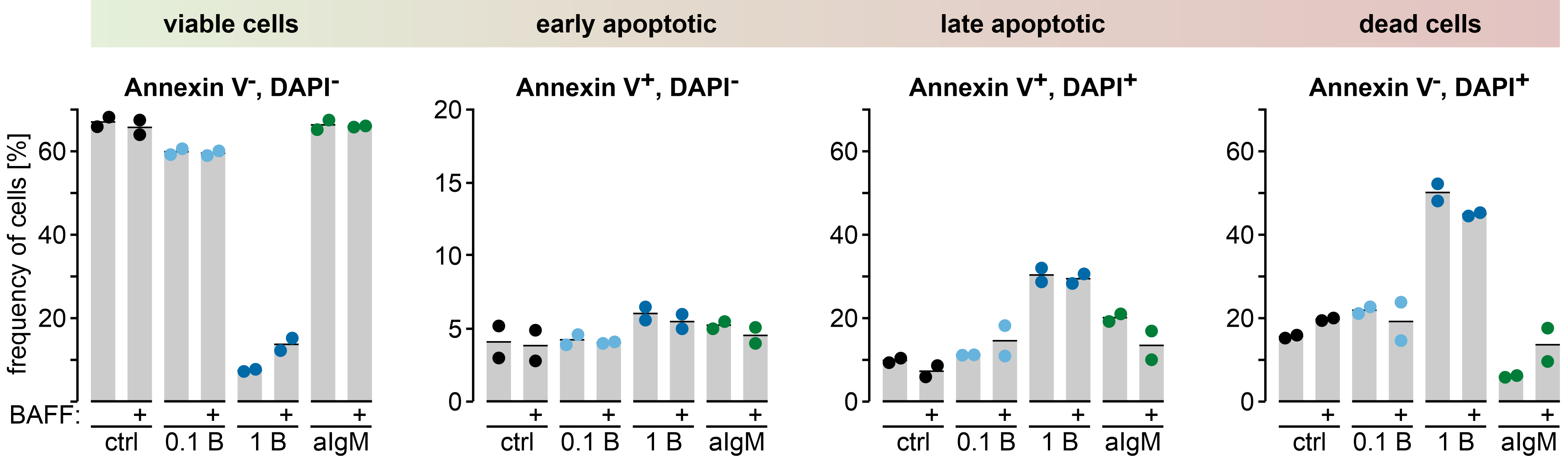


**Fig. S3 High BambL concentrations are cytotoxic and are not counteracted by BAFF**

Isolated B cells were exposed to 0.1 µg/mL BambL, 1.0 µg/mL BambL, 1.0 µg/mL anti-IgM (‘aIgM’) or left unstimulated (‘ctrl’). A second set of samples was supplemented with 25 ng/mL BAFF. After 16 h, the cells were collected and stained with Annexin V and DAPI to roughly characterize cell viability in flow-cytometric analyses. Gray bars are mean values of n = 2, circles represent individual data points.


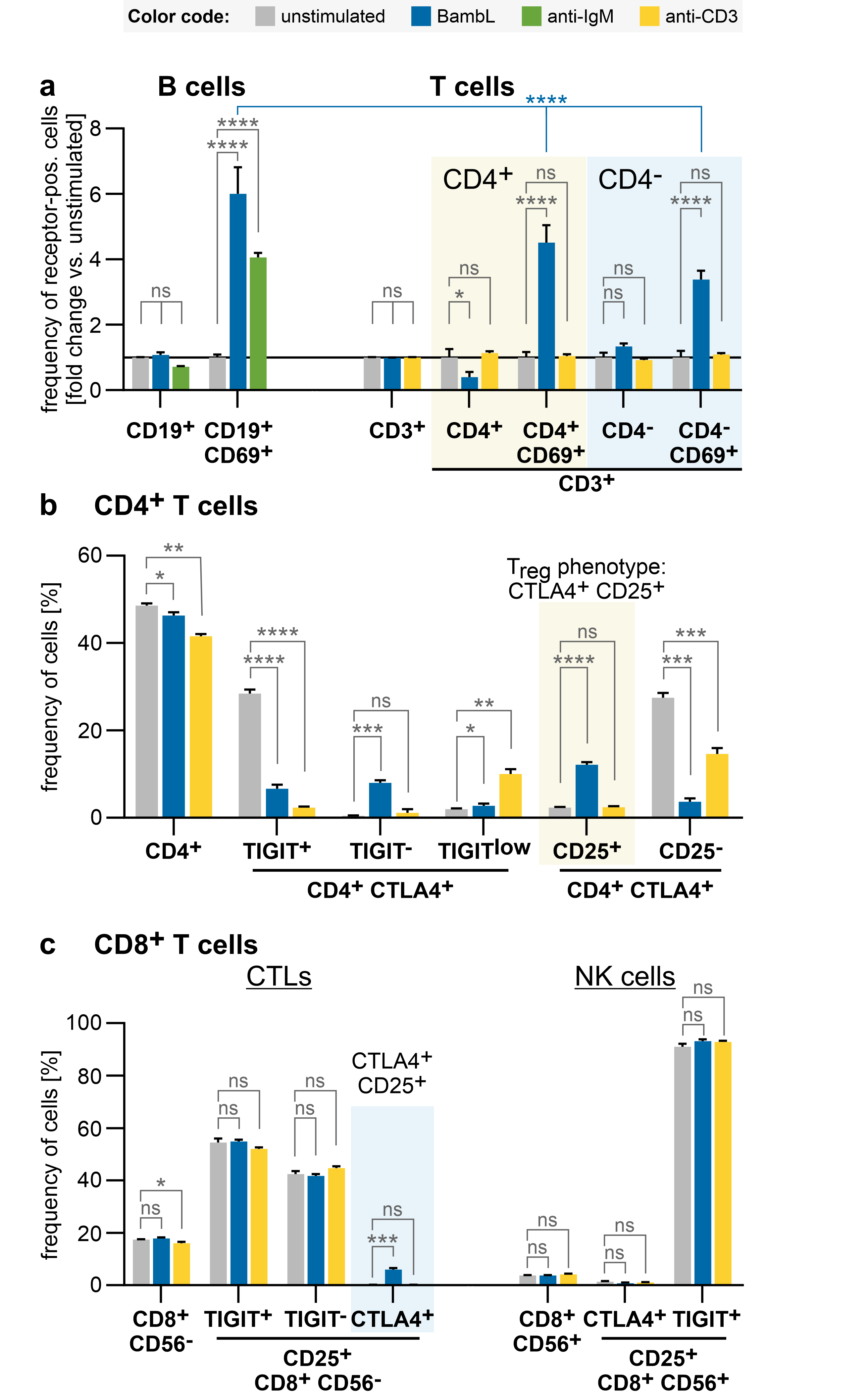


**Fig. S4 BambL activates human T cells and induces a CD25^+^ CTLA4^+^ T_reg_ phenotype.**

PBMCs were cultivated in presence of different stimuli for 16 h, then analyzed by flow cytometry. Statistical significance (ANOVA with Tukey’s correction): ns (p>0.05), ∗ (p≤0.05), ∗∗ (p≤0.01), ∗∗∗ (p≤0.001), ∗∗∗∗ (p≤0.0001).

**a BambL induces CD69 expression in both CD4^+^ and CD4^-^ T cells**. PBMCs were stimulated with 0.1 µg/mL BambL, 1 µg/mL anti‑IgM, or a surface coating of anti-CD3. Bars represent mean fold changes of viable cells versus unstimulated controls ± SD (n = 3). B cells displayed a stronger increase in the proportion of CD69^+^ cells after BambL exposure than either T cell subset.

**b CTLA4^+^ CD4^+^ T cells experience TIGIT downmodulation but CD25 upregulation**. PBMCs were stimulated with 0.1 µg/mL BambL or 1 µg/mL anti-CD3. Bars represent mean frequencies of viable cells ± SD (n = 4). Both stimuli caused a loss of TIGIT expression among CTLA4^+^ cells, but only BambL stimulated CD25 upregulation.

**c Cytotoxic T lymphocytes (CTLs) increase CTLA4^+^ CD25^+^ expression.** PBMCs were stimulated as in Fig. S3b (n = 4). BambL elicited an increase of the CTLA4^+^ CD25^+^ fraction among CTLs (CD8^+^ CD56^-^), but TIGIT levels remained unchanged. Receptor expression in natural killer cells (NK cells, CD8^+^ CD56^+^) was unaffected by BambL stimulation.


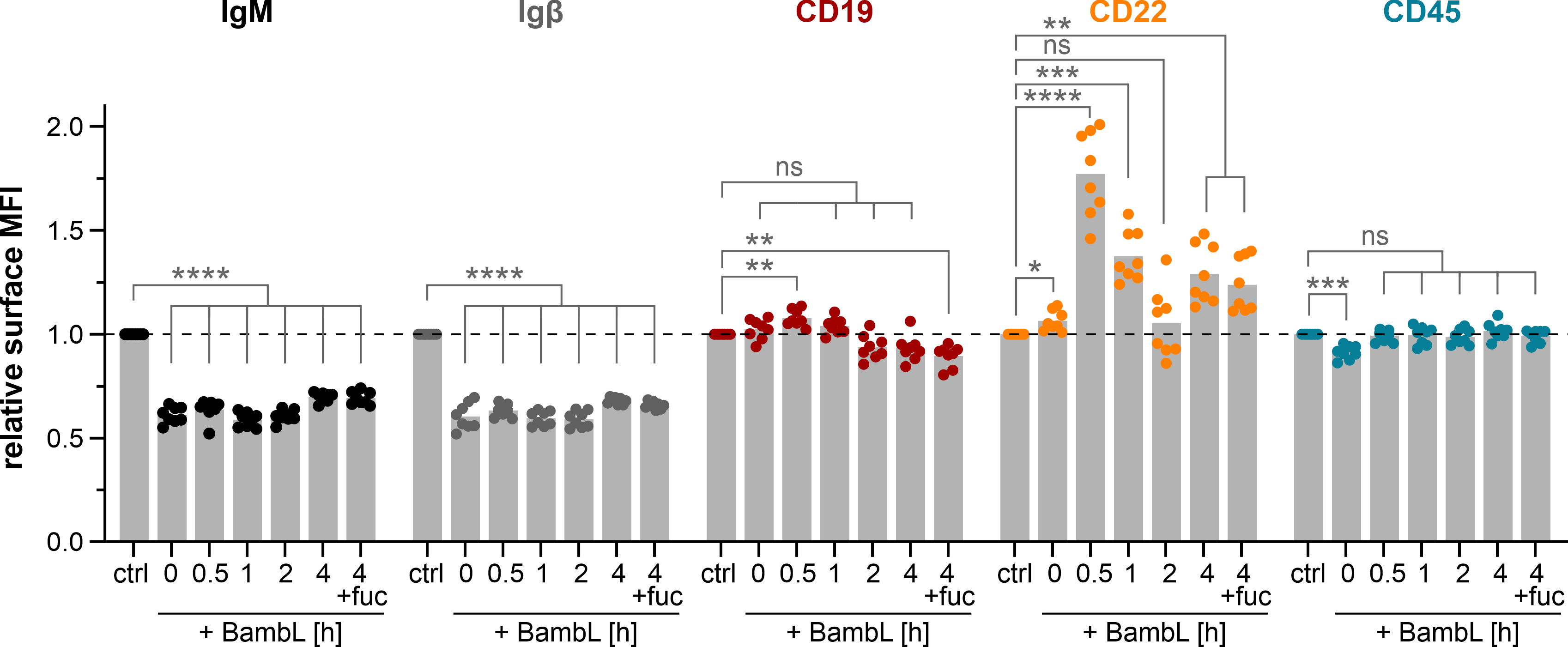


**Fig. S5 Anti-IgM depletes the BCR but not CD19 (supplement to Fig. 6b)**

Cell samples were prepared as for **Fig. 6b**, except that BambL was replaced with 1 µg/mL anti‑IgM F(ab’)_2_ (‘anti‑IgM’) during the stimulation step on ice. Briefly, Ramos cells were loaded with anti‑IgM on ice, washed and incubated in warm medium for indicated durations until flow cytometric analysis of surface proteins. As controls, cells were left unstimulated (‘ctrl’) or the treatment solution was supplemented with 10 mM fucose (‘4 h + fuc’). Bars are mean fold changes of geometric mean fluorescence intensities (MFI) relative to ‘ctrl’ samples (n = 8). Statistical significance (2‑way ANOVA with Dunnett’s correction): ns (p>0.05), ∗ (p≤0.05), ∗∗ (p≤0.01), ∗∗∗ (p≤0.001), ∗∗∗∗ (p≤0.0001).

**Tab. S1 SILAC-MS screen for BambL interaction partners on Ramos cells (supplement to Fig. 4a)**

Ranked by mean enrichment by BambL‑biotin (n=2). Top 120 hits with a mean enrichment greater than 4-fold. Selected proteins presented in **Fig. 4b** are highlighted in gray.

| **rank** | **Gene names** | **Protein names** | **enrichment over ctrl (log2)** | | |
| --- | --- | --- | --- | --- | --- |
|  |  |  | **exp 1** | **exp 2** | **Mean** |
| 1 | EVI2B | Protein EVI2B | 6.79 | 8.03 | 7.41 |
| 2 | SPN | Leukosialin | 7.84 | 6.93 | 7.39 |
| 3 | SELPLG | P-selectin glycoprotein ligand 1 | 7.44 | 6.19 | 6.82 |
| 4 | SLC2A5 | Solute carrier family 2, facilitated glucose transporter member 5 | 6.48 | 6.49 | 6.49 |
| 5 | ICAM2 | Intercellular adhesion molecule 2 | 6.99 | 5.98 | 6.49 |
| 6 | NCSTN | Nicastrin | 6.92 | 5.91 | 6.42 |
| 7 | CD22 | CD22 | 6.55 | 6.26 | 6.40 |
| 8 | PCDH9 | Protocadherin-9 | 6.74 | 6.02 | 6.38 |
| 9 | LNPEP | Leucyl-cystinyl aminopeptidase | 6.36 | 6.37 | 6.37 |
| 10 | ICAM3 | Intercellular adhesion molecule 3 | 6.32 | 6.35 | 6.33 |
| 11 | SLC1A4 | Neutral amino acid transporter A | 6.59 | 5.47 | 6.03 |
| 12 | SLC1A5 | Neutral amino acid transporter B(0) | 6.50 | 5.46 | 5.98 |
| 13 | INSR | Insulin receptor subunit alpha/beta | 7.01 | 4.50 | 5.76 |
| 14 | SLC2A1 | Solute carrier family 2, facilitated glucose transporter member 1 | 6.53 | 4.85 | 5.69 |
| 15 | PCDHGC3 | Protocadherin gamma-C3 | 7.60 | 3.70 | 5.65 |
| 16 | CD79B | BCR complex-associated protein beta chain, Ig beta | 5.94 | 5.35 | 5.65 |
| 17 | CD19 | B-cell antigen CD19 | 6.65 | 4.64 | 5.65 |
| 18 | SORL1 | Sortilin-related receptor | 6.80 | 4.44 | 5.62 |
| 19 | ATP1A1 | Sodium/potassium-transporting ATPase subunit alpha-1 | 5.46 | 5.54 | 5.50 |
| 20 | SLC12A2 | Solute carrier family 12 member 2 | 5.42 | 5.54 | 5.48 |
| 21 | SLC7A5 | Large neutral amino acids transporter small subunit 1 | 5.60 | 5.25 | 5.42 |
| 22 | CD48 | CD48 antigen | 3.49 | 7.17 | 5.33 |
| 23 | SLC3A2 | 4F2 cell-surface antigen heavy chain | 5.10 | 5.55 | 5.33 |
| 24 | GOLIM4 | Golgi integral membrane protein 4 | 5.86 | 4.64 | 5.25 |
| 25 | HLA-DRB1 | HLA class II histocompatibility antigen, DRB1-7/9 beta chains | 5.16 | 5.31 | 5.24 |
| 26 | SLC4A2 | Anion exchange protein 2 | 4.96 | 5.48 | 5.22 |
| 27 | HLA-DRA | HLA class II histocompatibility antigen, DR alpha chain | 5.21 | 5.01 | 5.11 |
| 28 | SDF4 | 45 kDa calcium-binding protein | 4.88 | 5.28 | 5.08 |
| 29 | HLA-DRB4 | HLA class II histocompatibility antigen, DR beta 4 chain | 5.17 | 4.87 | 5.02 |
| 30 | HLA-DPB1 | HLA class II histocompatibility antigen, DP beta 1 chain | 4.76 | 5.23 | 4.99 |
| 31 | SLAMF6 | SLAM family member 6 | 4.86 | 4.90 | 4.88 |
| 32 | PTPRC | Receptor-type tyrosine-protein phosphatase C | 5.88 | 3.85 | 4.87 |
| 33 | SLC4A7 | Sodium bicarbonate cotransporter 3 | 6.26 | 3.43 | 4.85 |
| 34 | SLC29A1 | Equilibrative nucleoside transporter 1 | 5.49 | 4.06 | 4.77 |
| 35 | CHP1 | Calcineurin B homologous protein 1 | 4.69 | 4.77 | 4.73 |
| 36 | SIT1 | Signaling threshold-regulating transmembrane adapter 1 | 3.87 | 5.59 | 4.73 |
| 37 | CD97 | CD97 antigen subunit alpha/beta | 6.80 | 2.63 | 4.72 |
| 38 | BST2 | Bone marrow stromal antigen 2 | 4.50 | 4.74 | 4.62 |
| 39 | ITGB1 | Integrin beta-1 | 4.64 | 4.55 | 4.59 |
| 40 | TMEM30A | Cell cycle control protein 50A | 6.06 | 3.06 | 4.56 |
| 41 | FAS | Tumor necrosis factor receptor superfamily member 6 | 4.84 | 3.97 | 4.41 |
| 42 | ATP1A3;ATP1A2 | Sodium/potassium-transporting ATPase subunits alpha-2/3 | 4.44 | 4.20 | 4.32 |
| 43 | ABCC4 | Multidrug resistance-associated protein 4 | 4.19 | 4.40 | 4.30 |
| 44 | ITGA4 | Integrin alpha-4 | 4.62 | 3.86 | 4.24 |
| 45 | IL4R | Interleukin-4 receptor subunit alpha | 4.87 | 3.60 | 4.23 |
| 46 | M6PR | Cation-dependent mannose-6-phosphate receptor | 2.63 | 5.79 | 4.21 |
| 47 | SYPL1 | Synaptophysin-like protein 1 | 4.59 | 3.80 | 4.20 |
| 48 | CD53 | Leukocyte surface antigen CD53 | 3.16 | 5.22 | 4.19 |
| 49 | BSG | Basigin | 3.63 | 4.57 | 4.10 |
| 50 | CD38 | ADP-ribosyl cyclase 1 | 4.75 | 3.44 | 4.10 |
| 51 | ATP1B3 | Sodium/potassium-transporting ATPase subunit beta-3 | 3.65 | 4.52 | 4.09 |
| 52 | MILR1 | Allergin-1 | 5.06 | 2.97 | 4.02 |
| 53 | LAMP1 | Lysosome-associated membrane glycoprotein 1 | 5.93 | 2.06 | 3.99 |
| 54 | LDLR | Low-density lipoprotein receptor | 3.09 | 4.83 | 3.96 |
| 55 | HYOU1 | Hypoxia-upregulated protein 1 | 4.00 | 3.70 | 3.85 |
| 56 | TGOLN2 | Trans-Golgi network integral membrane protein 2 | 2.10 | 5.42 | 3.76 |
| 57 | ABCC1 | Multidrug resistance-associated protein 1 | 4.37 | 3.03 | 3.70 |
| 58 | GLG1 | Golgi apparatus protein 1 | 5.38 | 1.97 | 3.68 |
| 59 | SLC7A1 | High affinity cationic amino acid transporter 1 | 3.64 | 3.64 | 3.64 |
| 60 | SLC25A3 | Phosphate carrier protein, mitochondrial | 3.33 | 3.86 | 3.60 |
| 61 | SLC25A3 | Intercellular adhesion molecule 1 | 3.93 | 3.21 | 3.57 |
| 62 | ALCAM | CD166 antigen | 3.48 | 3.42 | 3.45 |
| 63 | RPS2 | 40S ribosomal protein S2 | 3.72 | 3.14 | 3.43 |
| 64 | RPL9 | 60S ribosomal protein L9 | 4.01 | 2.85 | 3.43 |
| 65 | EMB | Embigin | 4.81 | 1.99 | 3.40 |
| 66 | ATP5L | ATP synthase subunit g, mitochondrial | 3.35 | 3.34 | 3.35 |
| 67 | RPS3 | 40S ribosomal protein S3 | 3.42 | 3.27 | 3.35 |
| 68 | ANKRD13A | Ankyrin repeat domain-containing protein 13A | 3.16 | 3.49 | 3.32 |
| 69 | B2M | Beta-2-microglobulin | 3.17 | 3.48 | 3.32 |
| 70 | GNPTAB | N-acetylglucosamine-1-phosphotransferase subunits alpha/beta | 2.71 | 3.89 | 3.30 |
| 71 | TMEM179B | Transmembrane protein 179B | 3.26 | 3.33 | 3.30 |
| 72 | RPL10;RPL10L | 60S ribosomal protein L10 | 3.61 | 2.74 | 3.18 |
| 73 | PTPRA | Receptor-type tyrosine-protein phosphatase alpha | 3.41 | 2.84 | 3.13 |
| 74 | VAMP8 | Vesicle-associated membrane protein 8 | 3.28 | 2.96 | 3.12 |
| 75 | RPS11 | 40S ribosomal protein S11 | 3.75 | 2.46 | 3.10 |
| 76 | CD22 | B-cell receptor CD22 | 3.32 | 2.80 | 3.06 |
| 77 | ATP5C1 | ATP synthase subunit gamma, mitochondrial | 2.91 | 3.17 | 3.04 |
| 78 | PPT1 | Palmitoyl-protein thioesterase 1 | 2.47 | 3.61 | 3.04 |
| 79 | TFRC | Transferrin receptor protein 1 | 3.14 | 2.91 | 3.02 |
| 80 | SCARB1 | Scavenger receptor class B member 1 | 4.56 | 1.48 | 3.02 |
| 81 | LRRC8D | Leucine-rich repeat-containing protein 8D | 3.41 | 2.63 | 3.02 |
| 82 | SLC38A5 | Sodium-coupled neutral amino acid transporter 5 | 3.32 | 2.61 | 2.97 |
| 83 | RPS7 | 40S ribosomal protein S7 | 3.40 | 2.45 | 2.93 |
| 84 | TMX1 | Thioredoxin-related transmembrane protein 1 | 2.98 | 2.86 | 2.92 |
| 85 | MAN2B1 | Lysosomal alpha-mannosidase | 2.90 | 2.91 | 2.91 |
| 86 | HLA-A | HLA class I histocompatibility antigen, A alpha chains | 2.65 | 3.14 | 2.90 |
| 87 | RPS15A | 40S ribosomal protein S15a | 3.25 | 2.53 | 2.89 |
| 88 | TMEM106B | Transmembrane protein 106B | 3.55 | 2.13 | 2.84 |
| 89 | RPS9 | 40S ribosomal protein S9 | 3.35 | 2.28 | 2.82 |
| 90 | RPL11 | 60S ribosomal protein L11 | 3.24 | 2.38 | 2.81 |
| 91 | RPS27;RPS27L | 40S ribosomal protein S27 | 3.00 | 2.62 | 2.81 |
| 92 | RPL28 | 60S ribosomal protein L28 | 3.44 | 2.16 | 2.80 |
| 93 | RPS3A | 40S ribosomal protein S3a | 3.10 | 2.38 | 2.74 |
| 94 | RPL19 | Ribosomal protein L19 | 3.06 | 2.41 | 2.73 |
| 95 | UBB;RPS27A;UBC;UBA52;UBBP4 | Ubiquitin-60S ribosomal protein L40 and 40S S27a, Polyubiquitin-B/C | 2.28 | 3.07 | 2.67 |
| 96 | RPS16 | 40S ribosomal protein S16 | 3.13 | 2.16 | 2.65 |
| 97 | CD74 | HLA class II histocompatibility antigen gamma chain | 2.04 | 3.25 | 2.65 |
| 98 | CD84 | SLAM family member 5 | 3.09 | 2.20 | 2.64 |
| 99 | RPL23 | 60S ribosomal protein L23 | 2.88 | 2.32 | 2.60 |
| 100 | ADAM17 | Disintegrin and metalloproteinase domain-containing protein 17 | 2.14 | 3.00 | 2.57 |
| 101 | SLC25A6;SLC25A4 | ADP/ATP translocases 1/3 | 2.90 | 2.09 | 2.50 |
| 102 | CDK1 | Cyclin-dependent kinase 1 | 2.64 | 2.29 | 2.46 |
| 103 | IGHM | Ig mu heavy chain C region | 2.10 | 2.81 | 2.45 |
| 104 | SLC25A5 | ADP/ATP translocase 2 | 2.83 | 2.01 | 2.42 |
| 105 | EHD1 | EH domain-containing protein 1 | 2.53 | 2.28 | 2.41 |
| 106 | LGALS9 | Galectin-9 | 3.04 | 1.73 | 2.39 |
| 107 | RPL13 | 60S ribosomal protein L13 | 2.48 | 2.28 | 2.38 |
| 108 | SLC44A2 | Choline transporter-like protein 2 | 2.54 | 2.17 | 2.35 |
| 109 | NAMPT;NAMPTL | Nicotinamide phosphoribosyltransferase | 2.54 | 2.13 | 2.34 |
| 110 | EIF4A3 | Eukaryotic initiation factor 4A-III | 2.92 | 1.66 | 2.29 |
| 111 | RPS14 | 40S ribosomal protein S14 | 2.32 | 2.24 | 2.28 |
| 112 | SNAP23 | Synaptosomal-associated protein 23 | 2.58 | 1.94 | 2.26 |
| 113 | RAN | GTP-binding nuclear protein Ran | 2.58 | 1.90 | 2.24 |
| 114 | ATP5A1 | ATP synthase subunit alpha, mitochondrial | 2.70 | 1.75 | 2.23 |
| 115 | MPZL1 | Myelin protein zero-like protein 1 | 0.99 | 3.41 | 2.20 |
| 116 | PHB2 | Prohibitin-2 | 2.53 | 1.71 | 2.12 |
| 117 | TOMM22 | Mitochondrial import receptor subunit TOM22 homolog | 1.85 | 2.35 | 2.10 |
| 118 | PKM | Pyruvate kinase isozymes M1/M2 | 2.10 | 1.98 | 2.04 |
| 119 | IGLC2;IGLC3;IGLC6;IGLC1 | Ig lambda-1/2/3/6 chain C regions | 1.82 | 2.26 | 2.04 |
| 120 | CAND1 | Cullin-associated NEDD8-dissociated protein 1 | 2.29 | 1.75 | 2.02 |

# Figure and Table Captions

**Fig. S1 Time course of DAPI sensitivity and cell counts in Lymphocytes gate (supplement to Fig. 2)**

B cells were isolated from PBMCs and cultivated in presence of three different BambL concentrations or left unstimulated for 4 days (n = 1 for each condition and time point). Samples were analyzed by flow cytometry after each day.

**a** **DAPI sensitivity.** Histograms of DAPI fluorescence; numbers indicate the frequencies of cells considered DAPI-positive in each sample.

**b** **Light scattering behavior.** Sideward (SSC) versus forward (FSC) scattering; numbers indicate frequencies in the lymphocytes gates.

**Fig. S2 BambL does not induce B cell proliferation at a non-toxic concentration**

Purified B cells were labeled with CellTrace Violet and cultivated in presence of 0.1 µg/mL BambL for 4 days. As controls, cells were stimulated with CpG oligonucleotides + BAFF or left unstimulated. Plots are representative of n = 4.

**a Proportion of viable B cells is similar to unstimulated controls.** Light scattering plots demonstrate no changes in viable B cell numbers upon BambL stimulation, unlike CpG + BAFF.

**b Fluorescence histogram of the proliferation dye remains unchanged.** BambL-treated cells present a single fluorescence peak, whereas CpG + BAFF induced 6 to 7 additional peaks at lower fluorescence intensity (representing 6 to 7 cell divisions).

**Fig. S3 High BambL concentrations are cytotoxic and are not counteracted by BAFF**

Isolated B cells were exposed to 0.1 µg/mL BambL, 1.0 µg/mL BambL, 1.0 µg/mL anti-IgM (‘aIgM’) or left unstimulated (‘ctrl’). A second set of samples was supplemented with 25 ng/mL BAFF. After 16 h, the cells were collected and stained with Annexin V and DAPI to roughly characterize cell viability in flow-cytometric analyses. Gray bars are mean values of n = 2, circles represent individual data points.

**Fig. S4 BambL activates human T cells and induces a CD25^+^ CTLA4^+^ T_reg_ phenotype.**

PBMCs were cultivated in presence of different stimuli for 16 h, then analyzed by flow cytometry. Statistical significance (ANOVA with Tukey’s correction): ns (p>0.05), ∗ (p≤0.05), ∗∗ (p≤0.01), ∗∗∗ (p≤0.001), ∗∗∗∗ (p≤0.0001).

**a BambL induces CD69 expression in both CD4^+^ and CD4^-^ T cells**. PBMCs were stimulated with 0.1 µg/mL BambL, 1 µg/mL anti‑IgM, or a surface coating of anti-CD3. Bars represent mean fold changes of viable cells versus unstimulated controls ± SD (n = 3). B cells displayed a stronger increase in the proportion of CD69^+^ cells after BambL exposure than either T cell subset.

**b CTLA4^+^ CD4^+^ T cells experience TIGIT downmodulation but CD25 upregulation**. PBMCs were stimulated with 0.1 µg/mL BambL or 1 µg/mL anti-CD3. Bars represent mean frequencies of viable cells ± SD (n = 4). Both stimuli caused a loss of TIGIT expression among CTLA4^+^ cells, but only BambL stimulated CD25 upregulation.

**c Cytotoxic T lymphocytes (CTLs) increase CTLA4^+^ CD25^+^ expression.** PBMCs were stimulated as in Fig. S3b (n = 4). BambL elicited an increase of the CTLA4^+^ CD25^+^ fraction among CTLs (CD8^+^ CD56^-^), but TIGIT levels remained unchanged. Receptor expression in natural killer cells (NK cells, CD8^+^ CD56^+^) was unaffected by BambL stimulation.

**Fig. S5 Anti-IgM depletes the BCR but not CD19 (supplement to Fig. 6b)**

Cell samples were prepared as for **Fig. 6b**, except that BambL was replaced with 1 µg/mL anti‑IgM F(ab’)_2_ (‘anti‑IgM’) during the stimulation step on ice. Briefly, Ramos cells were loaded with anti‑IgM on ice, washed and incubated in warm medium for indicated durations until flow cytometric analysis of surface proteins. As controls, cells were left unstimulated (‘ctrl’) or the treatment solution was supplemented with 10 mM fucose (‘4 h + fuc’). Bars are mean fold changes of geometric mean fluorescence intensities (MFI) relative to ‘ctrl’ samples (n = 8). Statistical significance (2‑way ANOVA with Dunnett’s correction): ns (p>0.05), ∗ (p≤0.05), ∗∗ (p≤0.01), ∗∗∗ (p≤0.001), ∗∗∗∗ (p≤0.0001).

**Tab. S1 SILAC-MS screen for BambL interaction partners on Ramos cells (supplement to Fig. 4a)**

Ranked by mean enrichment by BambL‑biotin (n=2). Top 120 hits with a mean enrichment greater than 4-fold. Selected proteins presented in **Fig. 4b** are highlighted in gray.
